# Comprehensive analysis of the cysteine-rich polycomb-like protein (CPP) gene family in peanut: insights into its expression patterns in abiotic stress responses

**DOI:** 10.3389/fpls.2026.1799353

**Published:** 2026-04-17

**Authors:** Hongzhan Liu, Zhuofan Wang, Manman Zheng, Siqiong Xu, Jiaxin Fu, Zehui Yin, Shiyu Wang, Chaoqiong Li, Kedong Xu, Yake Lei

**Affiliations:** 1 College of Life Science and Agronomy, Zhoukou Normal University, Zhoukou, Henan, China; 2 Field Observation and Research Station of Green Agriculture in Dancheng County, Zhoukou Normal University, Zhoukou, Henan, China; 3 College of Life Science, Northwest A&F University, Yangling, China; 4 Henan Key Laboratory of Crop Molecular Breeding and Bioreactor, Zhoukou Normal University, Zhoukou, Henan, China; 5 Oil Crops Research Institute, Zhoukou Academy of Agricultural Sciences, Zhoukou, Henan, China

**Keywords:** bioinformatics analysis, CPP gene family, droughtand salt stress, gene expression, synteny analysis

## Abstract

Peanut (*Arachis hypogaea*) is a crucial industrial crop whose production is severely limited by drought and salt stress. The CPP (cysteine-rich polycomb-like protein) gene family encodes cysteine-rich transcription factors with CXC domains that are involved in plant development and stress responses in addition to transcriptional regulation. However, their functional characterization in peanut remains largely unexplored. Here, the CPP gene family in peanut was systematically identified using bioinformatics approaches, after which its structural and functional attributes were comprehensively characterized. In total, 24 CPP genes were identified in the peanut genome; these genes were unevenly distributed across 15 chromosomes, with a relatively high density observed on chromosomes 9 and 16. All paralogs showed Ka/Ks less than 1, indicating strong purifying selection and functional conservation. A comparison of synteny revealed widespread collinearity of AhCPP genes across monocots and dicots, with AhCPP5 and AhCPP18 maintaining synteny in five species, highlighting their evolutionary stability. An analysis of cis-regulatory elements in AhCPP genes revealed the enrichment of diverse regulatory motifs, suggesting their potential roles in hormone signaling and stress responses in peanut. In addition, 116 putative miRNAs targeting 24 AhCPP genes were identified. Moreover, the transcriptomic analysis further revealed that AhCPP genes exhibited tissue- and stress-specific expression profiles in response to diverse abiotic stresses and hormonal stimuli. qRT-PCR analysis of six selected AhCPP genes suggested their potential involvement in the transcriptional regulation of drought and salt stress responses during the peanut seedling stage. Taken together, these findings provide a foundation for future functional investigations of AhCPPs for peanut breeding.

## Introduction

As the fifth-largest global oilseed crop, cultivated peanut (*Arachis hypogaea* L.) produces nutrient-rich edible oil and protein through its high-yield characteristics, delivering critical socioeconomic value to the global food system (Wang et al., 2025a). Drought and saline soil conditions represent the primary abiotic stresses during peanut growth, and impose significant constraints on peanut quality and potential yield (Sharma et al., 2016). Once plants perceive stress signals, transcription factors transduce and amplify these signals, thereby inducing the expression of stress-responsive genes (Yang et al., 2023). Compared with large transcription factor families such as MYB, NAC and WRKY, the cysteine-rich polycomb-like protein (CPP) family, although fewer in number, still plays a critical role in regulating development and stress adaptation in plants (Gu et al., 2025).

The CPP transcription factor, alternatively referred to as the TCX (tesmin/TSO1-like CXC protein) transcription factor, is absent in prokaryotes and fungi but is widely distributed in the animal and plant kingdoms and features one or two cysteine-rich CXC functional domains that bind to DNA to regulate target genes (Lu et al., 2013). The CXC domain, characterized by the conserved motif CXCX4CX3YCXCX6CX3CXCX2C separated by the conserved sequence RNPXAFXPK, is highly conserved in CPP genes (Andersen et al., 2007; Cvitanich et al., 2000; Hauser et al., 2000). Leveraging this conserved structure, genome-wide identification and analyses of the CPP gene family have been successfully conducted in multiple plant species, including *Arabidopsis thaliana* (*A. thaliana*) (Song et al., 2000), rice (*Oryza sativa*) (Yang et al., 2008), moso bamboo (*Phyllostachys edulis*) (Tan et al., 2024), tomato (*Solanum lycopersicum*) (Sun et al., 2023), and wheat (*Triticum aestivum*) (Ullah et al., 2022).

The first identified CPP gene was *TSO1*(*AtCPP5*) in *A. thaliana* (Hauser et al., 2000). Studies have confirmed that CPP plays important regulatory roles in the development of *Arabidopsis*, particularly in cell division and the flowering process (Sijacic et al., 2011). Additionally, researchers have found that *TSO1* regulates root and shoot development during seed germination by interacting with MYB-class proteins (Song et al., 2000; Wang et al., 2018). Using the latest B73 genome assembly (V5), researchers identified 12 ZmCPP genes in maize, which demonstrate differential expression patterns under abiotic stresses, as revealed by RT-qPCR analysis (Gu et al., 2025). Moreover, previous reports have indicated that four ZmCPP genes are upregulated in plants under drought stress (Song et al., 2016). A recent study revealed that the GSK3-like kinase SHAGGY-like kinase 1 (ZmSK1) negatively modulates drought tolerance in maize by phosphorylating the cysteine-rich transcriptional regulator *ZmCPP2* at Ser-250. This phosphorylation event disrupts the interaction between *ZmCPP2* and the promoter of *ZmSOD4*, a key gene encoding superoxide dismutase, thereby dampening antioxidant defense responses. In contrast, the unphosphorylated form of ZmCPP2 directly activates ZmSOD4 transcription, leading to increased antioxidant enzyme activity and consequently increasing drought tolerance (Yang et al., 2025). In soybean, CPP transcription factors exhibit tissue-specific expression in symbiotic rhizobial nodules and are involved in regulating the growth of symbiotic rhizobia (Cvitanich et al., 2000). Additionally, studies of the response of soybean roots to abiotic stresses have shown that the expression levels of most GmCPP genes are significantly upregulated in response to combined drought and high-temperature stresses, suggesting that these genes may be involved in the adaptive regulation of soybean roots to drought and high-temperature environments (Zhang et al., 2015). In wheat, members of the TaCPP gene family are highly expressed in leaves and spikes and exhibit differential expression patterns in response to abiotic stresses such as high temperature, drought, and salinity (Ullah et al., 2022). In cucumber, most members of the CsCPP gene family exhibit significant changes in their expression patterns following exposure to abiotic stresses such as salt stress, low temperature, drought, and ABA, indicating their potential involvement in adaptive responses to multiple abiotic stresses by regulating the stress response network (Zhou et al., 2018). A genomic study identified six SlCPP genes in tomato, most of which were upregulated in response to drought, salinity, and cold stresses, indicating their role in the environmental stress response (Sun et al., 2023).

Collectively, the results of previous studies have confirmed the close association between CPP genes and abiotic stresses. Despite the high sensitivity of peanut to abiotic stresses, its CPP gene family remains poorly studied. In this study, 24 members of the peanut CPP gene family were systematically analyzed, and their phylogeny, gene structures, conserved motifs, chromosomal locations, duplication events, collinearity, tissue expression, and stress-responsive profiles were characterized. These results provide a theoretical basis for functional studies of peanut CPP genes and insights for analyses of the CPP family in other species.

## Materials and methods

### Identification and analysis of the physicochemical properties of AhCPPs in peanut

We employed two complementary approaches to identify CPPs in peanut at the whole-genome level. First, known *A. thaliana* CPP protein sequences served as queries to search the PGR peanut genome database (http://peanutgr.fafu.edu.cn/) (Zhuang et al., 2019). Second, the CXC domain hidden Markov model (HMM) profile (PF03638) was obtained from Pfam (https://www.ebi.ac.uk/interpro/entry/pfam/PF03638/) and HMMER 3.0 was used to scan the peanut genome, and identify candidate proteins containing the CXC domain (e-value cutoff, 1e^-2^). Protein sequences obtained from both methods were combined, and duplicates were removed. The complete conserved domains of the putative AhCPPs were subsequently validated using SMART (Letunic and Bork, 2025) and PFAM (Mistry et al., 2021). For genes with multiple transcripts, the longest isoform was selected as the representative sequence. The genomic position, isoelectric point, aliphatic index, molecular weight, instability index, and grand average of hydropathicity (GRAVY) were queried and retrieved using the ExPASy online platform (Gasteiger et al., 2005). Transmembrane domains were predicted using the TMHMM (Krogh et al., 2001). The subcellular localization was predicted with the BUSCA web server (Savojardo et al., 2018). Proteins with an instability index below 40 are classified as stable, whereas those with an index above 40 are considered unstable, following the criteria established by Gamage et al. (2019).

### Secondary and tertiary structures prediction

The secondary structure and tertiary structure of AhCPP proteins were predicted using the SOPMA program (https://npsa-prabi.ibcp.fr/NPSA/npsa_sopm.html) and the SWISS-MODEL workspace on the ExPASy server (https://www.expasy.org), respectively.

### Phylogeny and classification of AhCPP gene family members in peanut

Full-length CPP sequences from –8 representative plant species whose diversity spans monocots and dicots, including Arabidopsis (Yang et al., 2008), pigeon pea (Nisar et al., 2022), moso bamboo (Tan et al., 2024), rice (Yang et al., 2008), tomato (Sun et al., 2023), sorghum (Du et al., 2025), soybean (Nisar et al., 2022), and peanut, were aligned using ClustalW (Thompson et al., 1994) with the default parameters. Additionally, the CPP protein information and download methods for the above species are provided in **Supplementary Table 1**. Based on this alignment, a phylogenetic tree was constructed in MEGA11 (Tamura et al., 2021) using the maximum likelihood (ML) method with 1000 bootstrap replicates and the default settings for the other parameters. Finally, the evolutionary tree underwent topological annotation and graphical optimization using iTOL v7 (https://itol.embl.de/).

### Analysis of conserved motifs and the gene structure

The genomic annotation (GFF3 file) of A. hypogaea was retrieved from the PGR database and used to delineate the gene structures of AhCPPs in TBtools (V2.324) (Chen et al., 2023). Conserved motifs were identified using MEME Suite (v5.5.8; https://meme-suite.org/meme/), with the maximum motif discovery set to 12. The resulting motif sequence logos were subsequently visualized and annotated using the TBtools (V2.324) integrated graphics module (Chen et al., 2023).

### Chromosomal localization and intraspecific collinearity analysis of AhCPPs

The whole-genome sequences of peanut were downloaded from the PGR database. The one-step MCScanX (Wang et al., 2012) and advanced Circos modules in TBtools (V2.324) were used to generate a chromosomal localization map and an intraspecific collinearity map of the AhCPP gene family.

### Analysis of gene duplication and synteny of AhCPPs

AhCPP duplications were detected using TBtools (V2.324). Interspecific collinearity between AhCPPs and four representative species (Arabidopsis, cucumber, wheat, and rice) was analyzed and visualized using the MCScanX module in TBtools (V2.324) (Chen et al., 2023).

### Prediction of cis−elements

Utilizing chromosomal coordinates from the peanut genome, the 2-kb promoter regions upstream of the CDS for AhCPP gene family members were extracted using the TBtools (V2.324) Gtf/Gff3 Sequence Extraction module. The cis-regulatory elements in these promoter sequences were predicted using the PlantCARE database (https://bioinformatics.psb.ugent.be/webtools/plantcare/html/). The distribution of identified cis-elements within AhCPP genes was subsequently visualized by employing the BioSequence Viewer in TBtools (V2.324) (Chen et al., 2023).

### Prediction of microRNAs targeting the AhCPP gene family in peanut

Putative miRNA target sites within AhCPP coding sequences (CDSs) were predicted using the psRNATarget platform (Dai et al., 2018) with the default parameters. The resulting miRNA–AhCPP regulatory networks were visualized using Cytoscape software (Shannon et al., 2003).

### Analysis of transcriptomic data for the peanut CPP gene family

The tissue-specific and stress-responsive expression profiles of the AhCPP genes were analyzed using peanut RNA-Seq data from PGR (http://peanutgr.fafu.edu.cn/Transcriptome.php). The expression values were log-transformed and visualized in heatmaps using TBtools (V2.324) (Chen et al., 2023).

### RNA isolation and qRT–PCR analysis of AhCPP genes

Seeds from the cultivated peanut Zhouhua11 were sterilized with 0.5% NaOCl, rinsed with ultrapure water, and germinated on sterile filter paper under controlled conditions (20 °C, 60% RH, 16/8-h photoperiod). Uniformly germinated seeds were cultured in a mixture of nutrient soil and vermiculite (25 °C, 60% RH, 16/8-h photoperiod). Upon reaching the 3–4-leaf stage, the seedlings were subjected to stress treatments involving root irrigation with either a 20% (w/v) PEG 6000 solution (drought simulation) (Chen et al., 2025; Li et al., 2025; Zhao et al., 2018) or 200 mmol/L NaCl (salt stress) (Zhang et al., 2020). Five days after treatment, samples of leaves, hypocotyls, and roots were collected and flash-frozen in liquid nitrogen for subsequent storage at -80 °C. High-quality total RNA was extracted from peanut tissues and reverse transcribed into first-strand cDNA using a commercial kit (RevertAid First Strand cDNA Synthesis Kit and DNase I; THERMO SCIENTIFIC). The sequences of the primers used for qRT–PCR (designed using Primer Premier, version 5.0; Premier, Canada) are detailed in **Supplementary Table 2**. Subsequently, qRT–PCR amplification was performed with Power SYBR^®^ Green PCR Master Mix on a real-time PCR system (Bio-Rad CFX Connect™). Moreover, three biological replicates were used for qRT-PCR, and each biological replicate was analyzed with triplicate technical replicates. Relative expression levels of the target genes were calculated using the 2^−ΔΔCT^ method (Schmittgen and Livak, 2008), employing *AhActin7* (AH20G09080.1) for data normalization. Data analysis and visualization, including Student’s, t-test (significance at p < 0.05) for two-group comparisons, were performed using GraphPad Prism 7 software (Mitteer et al., 2018).

## Results

### Identification of the peanut CPP gene family and analysis of the physicochemical properties of the proteins

Genome-wide identification revealed 24 AhCPP genes in cultivated peanut (*A. hypogaea* cv. PGR) through a homology screen using *Arabidopsis* AtCPP sequences and the PF03638 HMM profile, with subsequent validation using the SMART and Pfam databases. The analysis of the physicochemical properties using ExPASy-ProtParam revealed CDS lengths of 702–2,745 bp encoding 233–914 aa polypeptides, with molecular weights ranging from 26.03 to 102.36 kDa and theoretical pI values of 4.48–9.63. The aliphatic index (51.99–85.28) correlated with the variable thermostability, whereas the instability indices (33.82–65.54) indicated structural instability in 23 members (>40 threshold). The hydropathy analysis (GRAVY: −0.961 to 0.194) revealed 23 hydrophilic proteins and one hydrophobic member (AhCPP14). Notably, with the exception of AhCPP5 and AhCPP14, the vast majority of these proteins were devoid of transmembrane domains. Furthermore, the subcellular localization analysis confirmed the nuclear localization of every member of the AhCPP family (**Table 1**).

**Table 1:** CPP members in *Arachis hypogaea* and their physicochemical properties.

Gene name	Gene locus	CDS Length (bp)	AA^a^	MW^b^	pI^c^	II^d^	A.l.^e^	GRAVY^f^	TMD^g^	SLP^h^
AhCPP1	AH01G12710.1	1977	658	72.42	7.25	52.75	64.73	-0.613	0	Nucleus
AhCPP2	AH03G20660.1	1797	598	64.62	8.09	57.5	65.74	-0.557	0	Nucleus
AhCPP3	AH05G14650.1	1812	603	66.91	5.69	51.58	60.8	-0.754	0	Nucleus
AhCPP4	AH05G26270.1	1788	595	63.93	7.93	53.69	61.85	-0.641	0	Nucleus
AhCPP5	AH06G04470.2	2742	913	102.36	7.86	51.35	67.31	-0.73	1	Nucleus
AhCPP6	AH08G24840.1	1074	357	39.57	4.97	45.38	57.39	-0.833	0	Nucleus
AhCPP7	AH08G26550.1	1569	522	58.39	4.88	55.18	63.31	-0.86	0	Nucleus
AhCPP8	AH09G11020.1	2553	850	95.19	7.76	46.73	65.42	-0.699	0	Nucleus
AhCPP9	AH09G15340.1	1107	368	41.55	5.11	56.64	53.29	-0.836	0	Nucleus
AhCPP10	AH09G26110.1	2241	746	81.55	6.32	64.05	67.37	-0.697	0	Nucleus
AhCPP11	AH10G21670.1	1515	504	55.94	6.53	50.55	62.1	-0.629	0	Nucleus
AhCPP12	AH11G12790.1	2085	694	76.74	7.24	53.58	65.61	-0.587	0	Nucleus
AhCPP13	AH13G23400.1	1800	599	64.83	8.21	56.33	66.11	-0.56	0	Nucleus
AhCPP14	AH14G06350.1	702	233	26.03	9.63	33.82	85.28	0.194	2	Nucleus
AhCPP15	AH14G22800.1	879	292	32.60	9.51	48.55	73.42	-0.216	0	Nucleus
AhCPP16	AH15G08250.2	1869	622	69.14	5.62	57.37	62.86	-0.699	0	Nucleus
AhCPP17	AH15G29200.1	1788	595	63.79	8.09	51.92	62.67	-0.61	0	Nucleus
AhCPP18	AH16G07800.1	2583	860	96.50	7.59	49.67	64.31	-0.795	0	Nucleus
AhCPP19	AH16G36030.1	2736	911	99.08	6.15	63.75	64.76	-0.674	0	Nucleus
AhCPP20	AH16G46810.1	2745	914	99.37	6.09	65.54	64.76	-0.669	0	Nucleus
AhCPP21	AH18G29450.1	1044	347	38.69	5.53	49.42	51.99	-0.961	0	Nucleus
AhCPP22	AH18G31830.1	1743	580	64.45	4.89	64.78	61.69	-0.851	0	Nucleus
AhCPP23	AH19G42020.1	2244	747	81.72	6.32	63.59	67.54	-0.695	0	Nucleus
AhCPP24	AH20G28460.1	1815	604	67.11	7.96	55.77	66.03	-0.612	0	Nucleus

^a^Length of the amino acid sequence. ^b^Molecular weight of the amino acid sequence. ^c^Isoelectric point of the AhCPP proteins. ^d^Instability Index. ^e^Aliphatic Index. ^f^GRAVY (Grand Average of Hydropathicity) the hydrophilic or hydrophobic nature of the protein; positive values indicate hydrophobic proteins, while negative values indicate hydrophilic proteins. ^g^Number of transmembrane domains, as predicted by the TMHMM server (https://services.healthtech.dtu.dk/services/TMHMM-2.0/). ^h^Protein subcellular localization prediction by the BUSCA web server (https://busca.biocomp.unibo.it/).

### Prediction of the secondary/tertiary structures of AhCPPs

The secondary structures of AhCPP proteins, which were predicted with the SOPMA online tool, are composed of α-helices, β-turns, extended strands, and random coils (**Supplementary Table 3**). An analysis of the secondary structure revealed that α-helices and random coils are the predominant elements in AhCPP proteins, comprising 13.46–46.78% and 43.35–83.48% of their structures, respectively. In contrast, β-turns and extended strands constitute only minor and variably distributed components. Notably, AhCPP14 exhibited the highest α-helix content (46.78%), whereas that of AhCPP20 was the lowest (13.46%). These structural characteristics, dominated by flexible random coils and α-helices, may underpin the molecular stability and functional diversity of the AhCPP family. Furthermore, the tertiary structures predicted using the ExPASy tool indicated that all AhCPPs adopt a generally similar fold dominated by α-helices and random coils, which is consistent with the results of the secondary structure analysis. Despite this overall similarity, discernible structural distinctions exist between different AhCPPs, potentially underlying their functional diversification (**Supplementary Figure S1**).

### Phylogenetic analysis of AhCPPs in A. hypogaea

**Figure 1:**
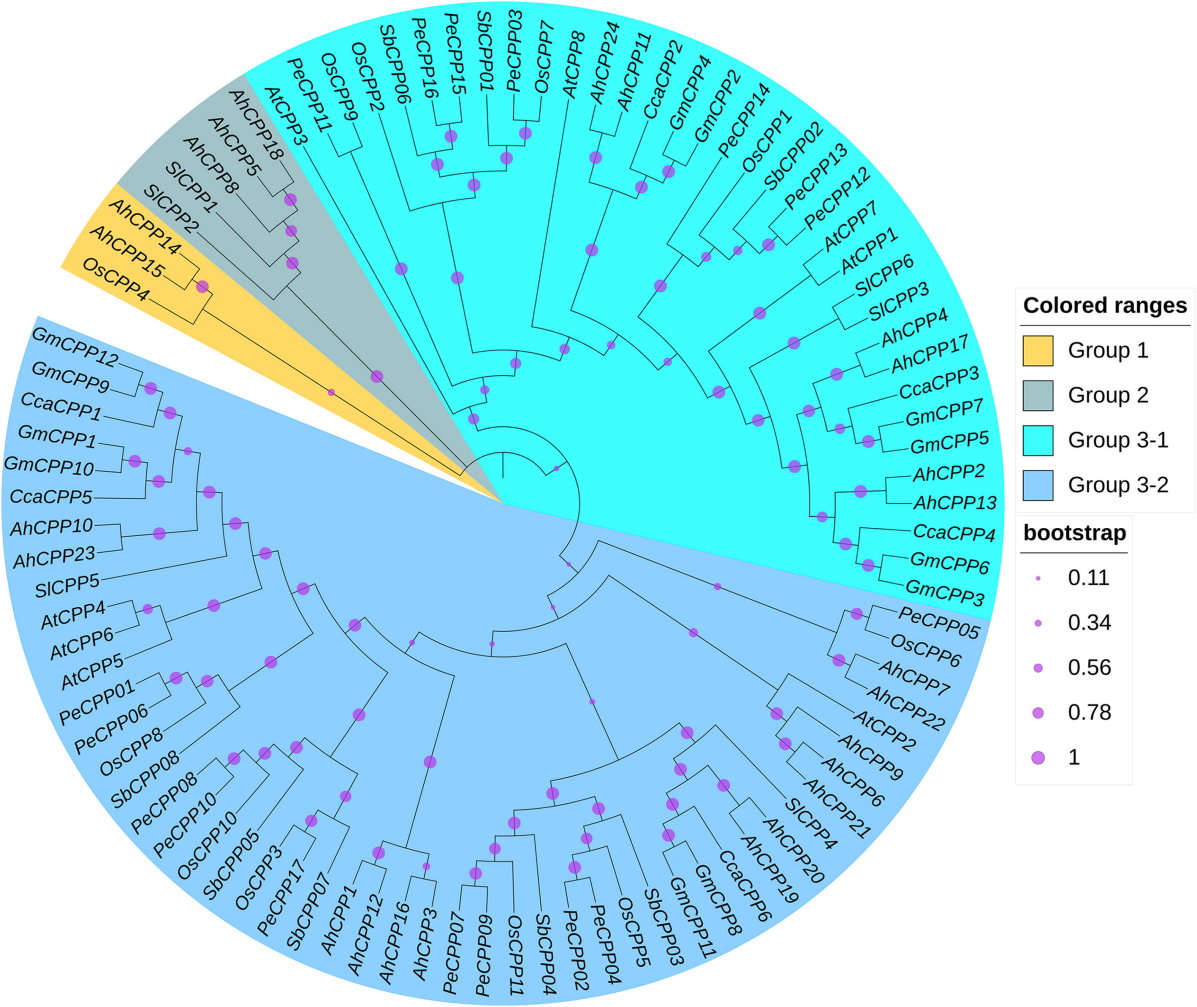
Phylogenetic analysis of CPP homologs across different species. A maximum likelihood phylogenetic tree of CPPs was constructed using –92 protein sequences: 8 from *Arabidopsis thaliana* (AtCPP), 24 from *Arachis hypogaea* (AhCPP), 6 from *Cajanus cajan* (CcaCPP), 17 from *Phyllostachys edulis* (PeCPP), 11 from *Oryza sativa* (OsCPP), 6 from *Solanum lycopersicum* (SlCPP), 12 from soybean, and 8 from *Sorghum bicolor* (SbCPP). The tree is divided into three major groups (color-coded), with group 3 further subdivided into two subgroups. The size of the purple circles on the branches corresponds to the bootstrap support values.

A maximum-likelihood phylogenetic tree was generated with CPP homologs from eight species: peanut (24), *Arabidopsis* (8), pigeon pea (6), moso bamboo (17), rice (11), tomato (6), sorghum (8), and soybean (12) (**Figure 1**). Based on phylogenetic analysis with previously characterized CPP proteins from *Arabidopsis thaliana* and *Oryza sativa*, the 92 CPPs identified in this study were classified into three main groups, consistent with the subgroup classification criteria established for CPP subfamilies in model plants. Both Group 1 and Group 2 contained only a few peanut CPPs and no *Arabidopsis* CPPs. Group 1 included one rice CPP (OsCPP4) together with two peanut AhCPPs (AhCPP14 and AhCPP15), while Group 2 contained two tomato CPPs (SlCPP1 and SlCPP2) along with three peanut AhCPPs (AhCPP5, AhCPP8, and AhCPP18). Group 3 (the largest group) was split into two subgroups: Group 3-1 and Group 3-2. In subgroup 3-1, six AhCPPs clustered with *Arabidopsis* AtCPP1, 3, 7, and 8. Subgroup 3- 2 contained 13 AhCPPs grouped with AtCPP2, 4, 5, and 6. Notably, several AhCPPs (e.g., AhCPP10 and AhCPP23) showed strong evolutionary connections with high bootstrap values (0.996-1) to dicot proteins, including pigeon pea CcaCPP1/CcaCPP5 and tomato SiCPP5 and soybean GmCPP1/9/10/12. This pattern may suggest shared evolutionary paths and indicates close genetic relationships among these species (**Figure 1**).

### Conserved motifs, domains, and sequence structure features of AhCPP members

**Figure 2:**
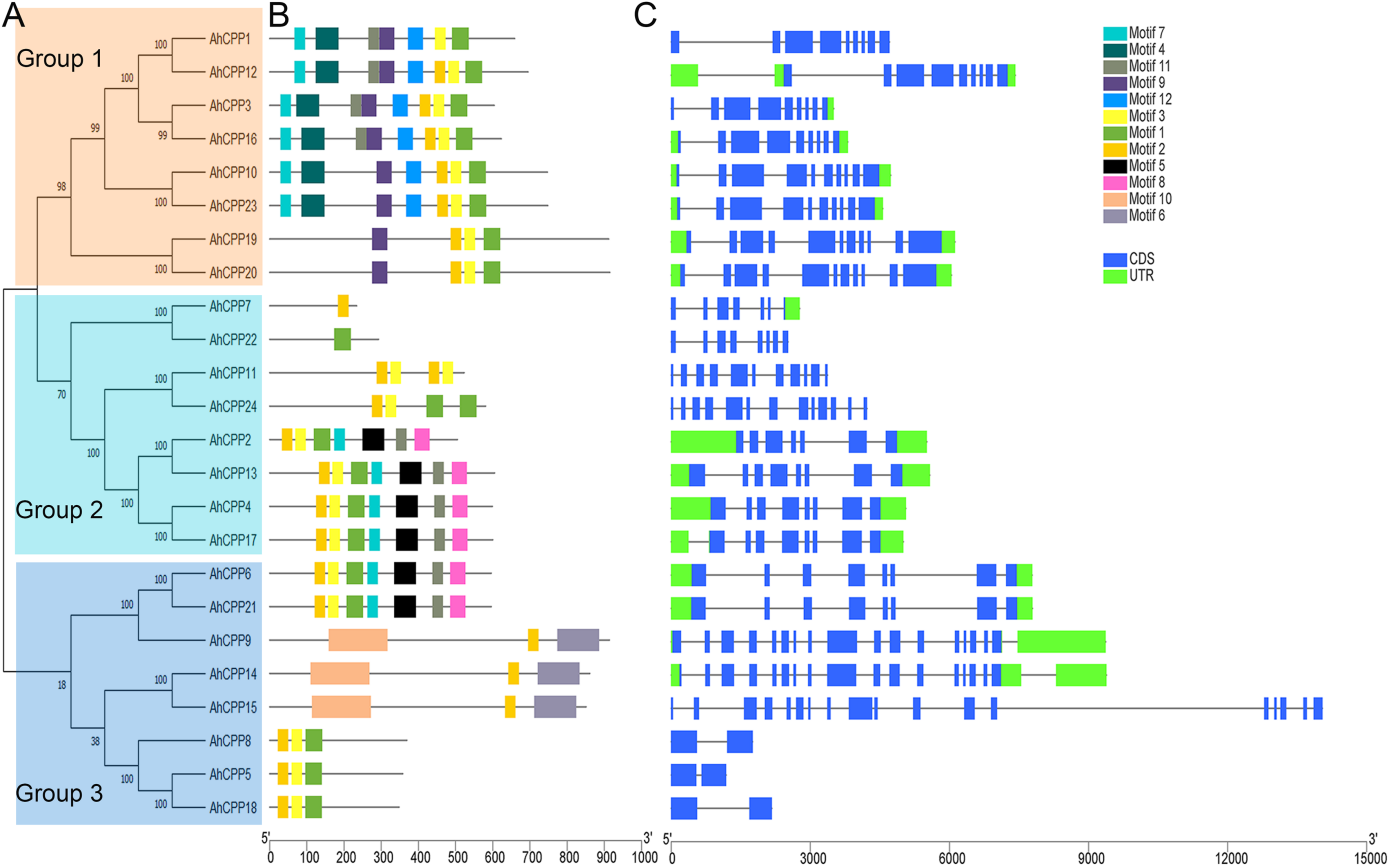
Conserved motifs and gene structure of AhCPPs in peanut. **(A)** The phylogenetic tree was generated using the methodology applied in **Figure 1**. Different colors represent different subgroups. **(B)** Colored boxes represent various motifs as indicated in the upper-right legend. Black lines denote nonconserved sequences from the MEME analysis. **(C)** Colored boxes depict various sequence elements (CDSs and UTRs), with a legend provided in the upper right corner. Black lines denote introns.

The conserved motifs of AhCPPs were characterized using the MEME platform to explore their functional domains. Using the MEME tool, twelve motifs were analyzed in the 24 proteins, with the minimum and maximum motif widths set at 6 to 200. The results of the MEME analysis were consistent with the results of the phylogenetic tree (**Figures 2A, B**). Analysis of the conserved domains in the amino acid sequences revealed that all the AhCPPs contain conserved CXC domains (**Supplementary Figure S2**), with numbers ranging from 1 to 3. Additionally, Motifs 1, 2, and 3 are present in the vast majority of members. Notably, AhCPP7, 9, 14, and 15 lack Motifs 1 and 3 but retain Motif 2 (**Figure 2B**). These findings suggest that these motifs are relatively conserved during the evolution of AhCPPs. The analysis of the structural characteristics of the peanut AhCPP genes shows that the number of exons ranges from 2 to 18 (**Figure 2C**). *AhCPP15* contains 18 exons and lacks UTR regions; *AhCPP5*, *8*, and *18* have the fewest exons (two exons) and lack UTR regions (**Figure 2C**). Among the 24 AhCPP genes, 16 possess UTRs, whereas the other 8 lack these regions. Additionally, AhCPP genes clustered on the same phylogenetic branch exhibit similar intron–exon distributions and structures.

### Chromosomal localization and analysis of the intraspecific collinearity of AhCPP genes

**Figure 3:**
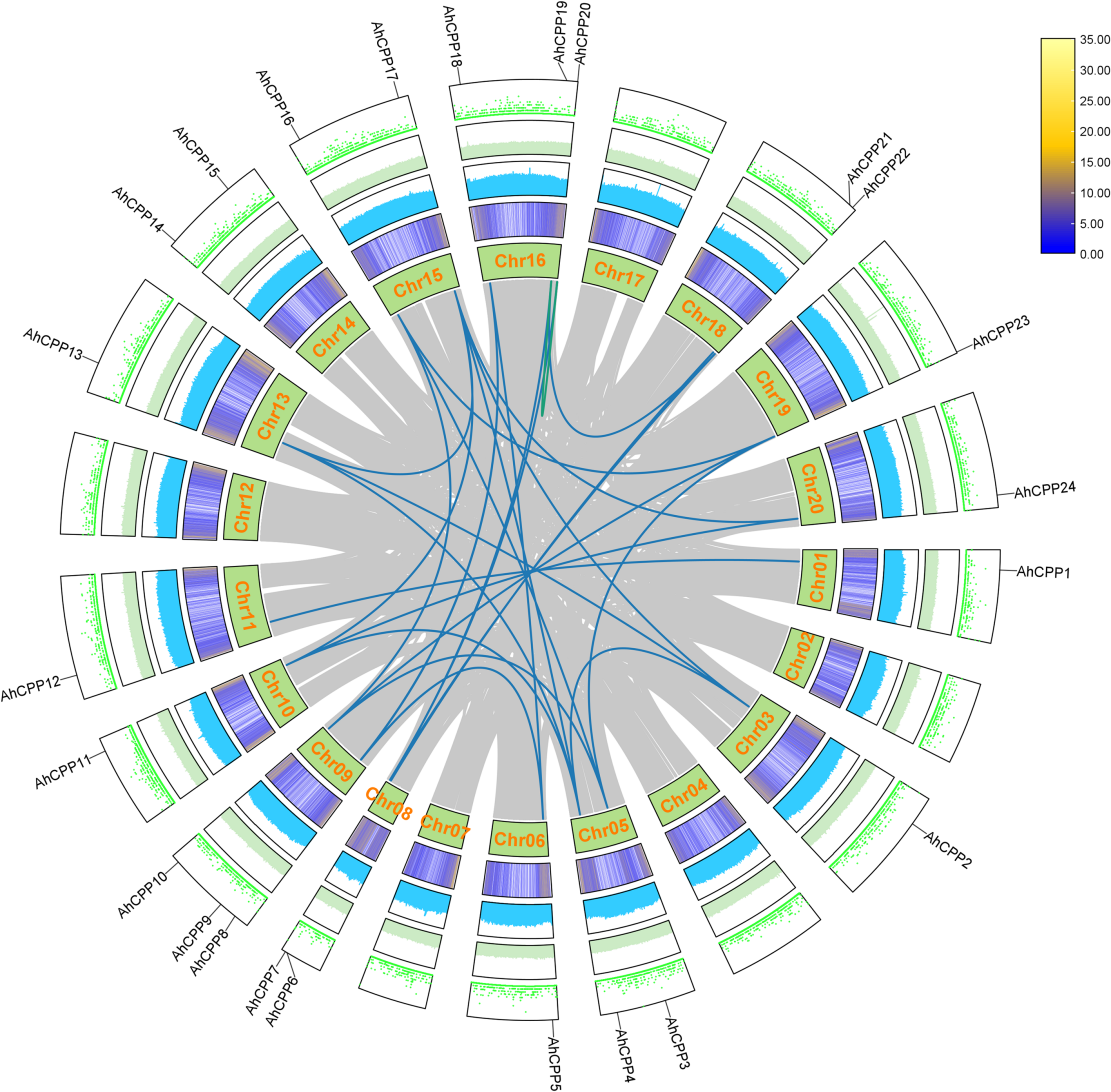
Circos plot illustrating the genomic distribution and duplication relationships of AhCPP genes in peanut. The circular tracks from inside to outside represent the chromosomal architecture, gene density, GC content, GC skew, and N ratio. AhCPP genes are differentially distributed across peanut chromosomes, with the chromosome numbers labeled in orange on the corresponding chromosomal ideograms. Homologous AhCPP gene pairs derived from segmental duplication are connected by blue lines, whereas those resulting from tandem duplication are linked by green lines. All AhCPP gene names are annotated on the outermost track. The gray lines in the background represent homologous relationships among other genes in the peanut genome.

AhCPP genes were not distributed on peanut chromosomes 2, 4, 7, 12, or 17 and were unevenly distributed across the remaining 15 chromosomes. The number of AhCPP genes (three each) was greatest on chromosomes 9 and 16, while chromosomes 5, 8, 14, 15, and 18 each contained two genes. One AhCPP gene was detected on each of chromosomes 1, 3, 6, 10, 11, 13, 19, and 20 (**Figure 3**). Further analysis revealed a tandem duplication event involving *AhCPP19* and *AhCPP20* in the AhCPP gene family. With the exception of AhCPP9, AhCPP14, and AhCPP15, segmental duplication events were observed in all the other gene family members, accounting for 27 collinear gene pairs. Among these genes, *AhCPP17* exhibited the highest occurrence of segmental duplication collinearities (5). In contrast, the lowest frequency was observed for AhCPP1, AhCPP5, AhCPP6, AhCPP12, and AhCPP21, each of which displayed only one collinearity (**Figure 3**). Moreover, the ratio of nonsynonymous (Ka) to synonymous (Ks) substitutions, which indicates selection pressure on coding sequences, was calculated (**Supplementary Table 4**). The Ka/Ks analysis revealed purifying selection acting on all the peanut *AhCPP* genes (values <1.0), with a maximum ratio of 0.696 observed for the paralogous pair “AhCPP6/AhCPP21” (**Supplementary Figure S3**; **Supplementary Table 4**).

### Analysis of the synteny of AhCPP genes between peanut and other plant species

**Figure 4:**
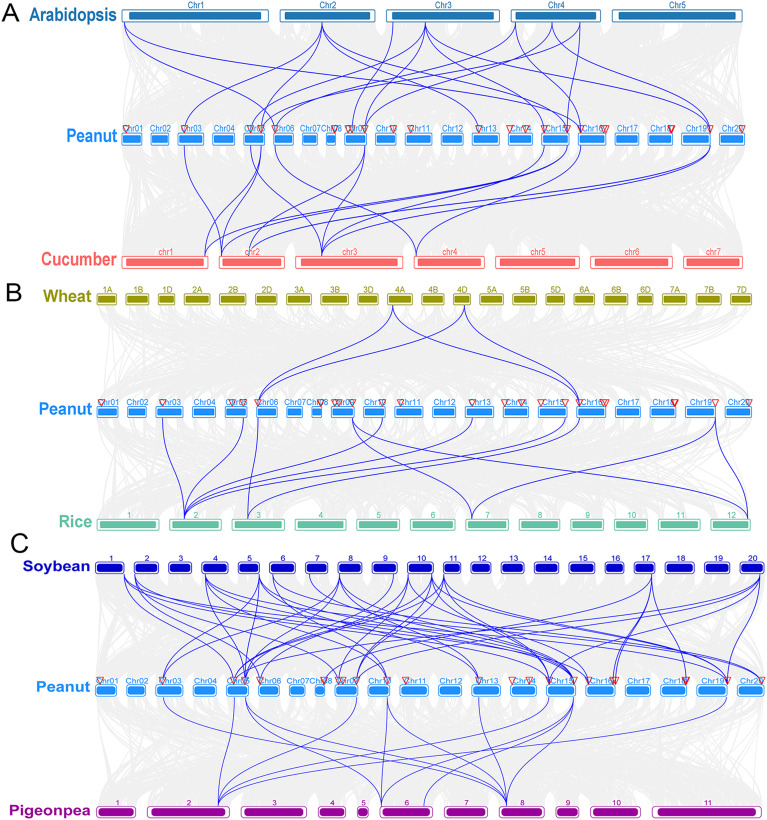
Analysis of the synteny of CPP genes between cultivated peanuts and six other plant species. **(A)** Interspecific synteny analysis among cultivated peanut, *Arabidopsis*, and cucumber, illustrating the evolutionary relationships of CPP genes in dicot plants. **(B)** Interspecific synteny analysis among cultivated peanut, wheat, and rice, displaying the evolutionary relationships of CPP genes in monocot plants. **(C)** Interspecific synteny analysis among cultivated peanut, soybean, and pigeonpea, displaying the evolutionary relationships of CPP genes in legume species. The gray lines represent all syntenic gene pairs in the genome, whereas the blue lines highlight syntenic CPP gene pairs.

Syntenic maps between peanut and six other plants were constructed in this study to elucidate the evolutionary mechanisms of the AhCPP gene family (**Figure 4**; **Supplementary Table 5**). The results showed that peanut (*A. hypogaea*) shares 17, 13, 4, 12, 41 and –13 pairs of orthologous genes with Arabidopsis (*A. thaliana*), cucumber (*C. sativus*), wheat (*T. aestivum*), rice (*O. sativa*), soybean (*G. max*), and pigeonpea (*C. cajan*), respectively. The results indicate significant syntenic conservation between peanut and dicot species, particularly with soybean, suggesting a potential link to their close phylogenetic relationships within the legume family and possible paleopolyploidization history. Chromosomes 5 and 15 of peanut each contain three corresponding pairs of orthologous genes with both *Arabidopsis* and cucumber. Cucumber orthologs are distributed on chromosomes 1–4, with no syntenic loci detected on other chromosomes (**Figure 4A**; **Supplementary Table 5**). The comparative synteny analysis revealed the substantial conservation of collinear loci between peanut AhCPP genes and both monocot and dicot species, with the singular exception of an orthologous gene pair connecting peanut chromosome 10 and rice chromosome 2, which was not detected in dicot systems. Among the investigated species, wheat possessed the fewest collinear gene pairs (4 pairs) localized exclusively to chromosomes 4A and 4D, while rice orthologs clustered on chromosomes 2, 3, 7, and 12 (**Figure 4B**; **Supplementary Table 5**). Extensive synteny was revealed between peanut and soybean (41 orthologous pairs), with multiple AhCPP genes corresponding to several soybean loci (e.g., *AhCPP3*, *4*, *16*, *17*, *23* mapped to four loci, respectively), reflecting the impact of legume-specific whole-genome duplication events. In contrast, synteny with pigeonpea was more limited (13 pairs), concentrated primarily on chromosomes 2, 6, and 8. Genes exhibiting broad synteny with soybean (e.g., *AhCPP3*, *4*, *16*, and *17*) possessed fewer orthologs in pigeonpea, suggesting lineage-specific gene loss. Specifically, nine AhCPP genes (*AhCPP2*, *3*, *4*, *10*, *11*, *13*, *16*, *17*, *23*) showed syntenic conservation with both legumes, while eight (*AhCPP5*, *7*, *8*, *18*, *19*, *20*, *22*, *24*) were exclusively syntenic with soybean (**Figure 4C**; **Supplementary Table 5**). Notably, *AhCPP5* (chromosome 6) and *AhCPP18* (chromosome 16) maintained synteny across five diverse species (*Arabidopsis*, cucumber, soybean, rice, and wheat) but were absent in pigeonpea (**Figure 4**; **Supplementary Table 5**). This suggests they may represent ancestral core CPP genes retained since early angiosperm evolution but lost specifically in the pigeonpea lineage. Overall, these findings underscore high syntenic conservation between peanut and soybean, stemming from their close phylogenetic proximity and shared paleopolyploidization history.

### Analysis of the cis-elements of the peanut CPP gene family

**Figure 5:**
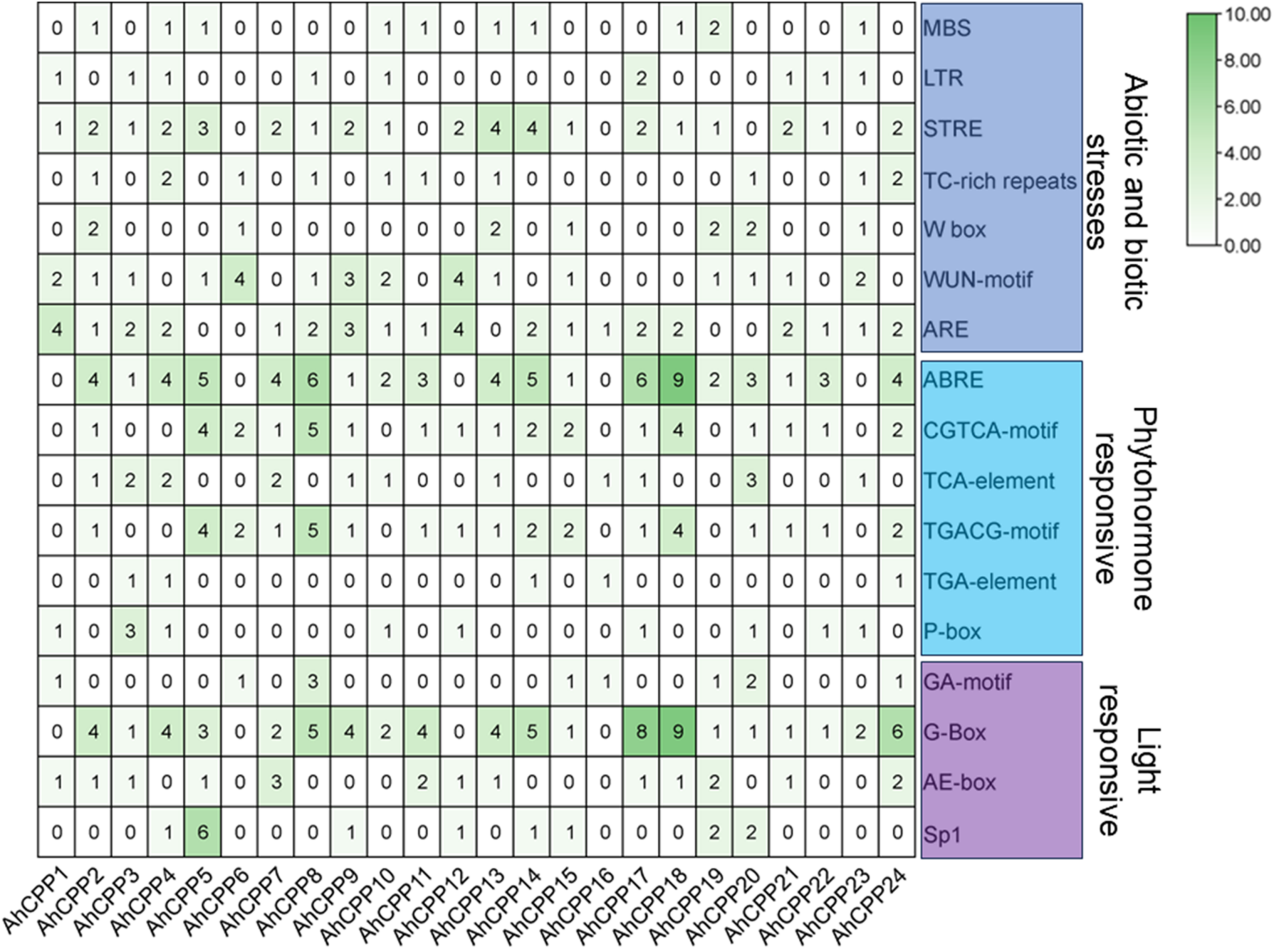
Distribution of cis-elements in the promoters of AhCPP genes. Putative cis-elements were identified within the 2-kb promoter regions upstream of the translation start sites of the peanut CPP genes. Values in the heatmap represent the number of occurrences of each cis−regulatory element in the promoter region of the corresponding gene.

The cis-regulatory elements within the 2,000 bp promoter regions upstream of each member were analyzed to elucidate potential transcriptional regulatory mechanisms involving the peanut CPP gene family. The functional annotation of AhCPP promoter regions using TBtools revealed 17 cis-acting elements (**Figure 5** and **Supplementary Figure S4**) classified into three functional categories: (1) stress-responsive elements (MBS, LTR, STRE, TC-rich repeats, W-box, WUN-motif, ARE); (2) hormone signaling-associated elements [ABRE (ABA-responsive), CGTCA-motif (MeJA-responsive), TGACG-motif (MeJA-responsive), TCA-element (salicylic acid-responsive), TGA-element (auxin-responsive), and P-box (gibberellin-responsive)]; and (3) light-responsive elements (GA-motif, G-box, AE-box, and SP1) (**Figure 5** and **Supplementary Figure S4**). Analysis of the 24 AhCPP gene promoters identified 409 cis-elements with distinct spatial distributions (**Figure 5**). Phytohormone-responsive elements constituted the largest category (161), with ABRE being the most frequent (79.2%; present in 19/24 genes). The frequent coupling of ABRE with the light-responsive G-Box implies a functional integration of ABA and light signaling in AhCPP regulation. Similarly, the prevalence of MeJA-responsive motifs (CGTCA/TGACG), found in 70.8% of the genes, suggests regulatory crosstalk between the MeJA and ABA pathways. Regarding stress-responsive components, ARE and STRE predominated, being present in 79.2% (19/24) and 75.0% (18/24) of the promoters, with 35 and 33 copies, respectively. Notably, the cis-element density was highly variable, with AhCPP8 and AhCPP16 containing 35 and 4 elements, respectively.

These findings suggest that AhCPPs may critically coordinate stress adaptation and developmental processes in peanut.

### Identifying miRNAs targeting AhCPP genes throughout the genome

**Figure 6:**
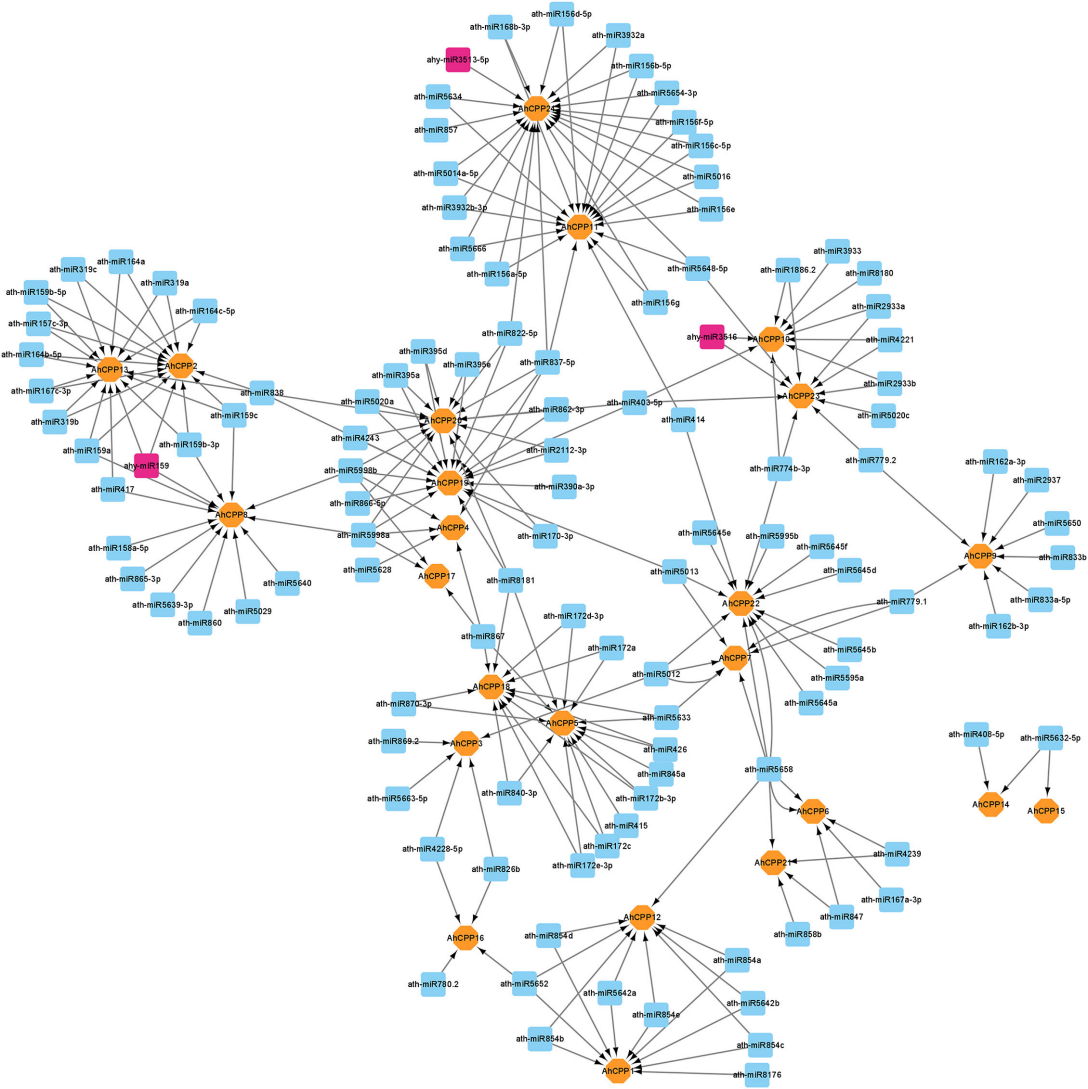
miRNAs targeting AhCPP genes in peanut. The miRNA target network of AhCPP genes is depicted, where yellow circles represent AhCPP genes, blue squares denote predicted miRNAs from *Arabidopsis thaliana*, and purple squares indicate predicted miRNAs from *Arachis hypogaea*.

**Figure 7:**
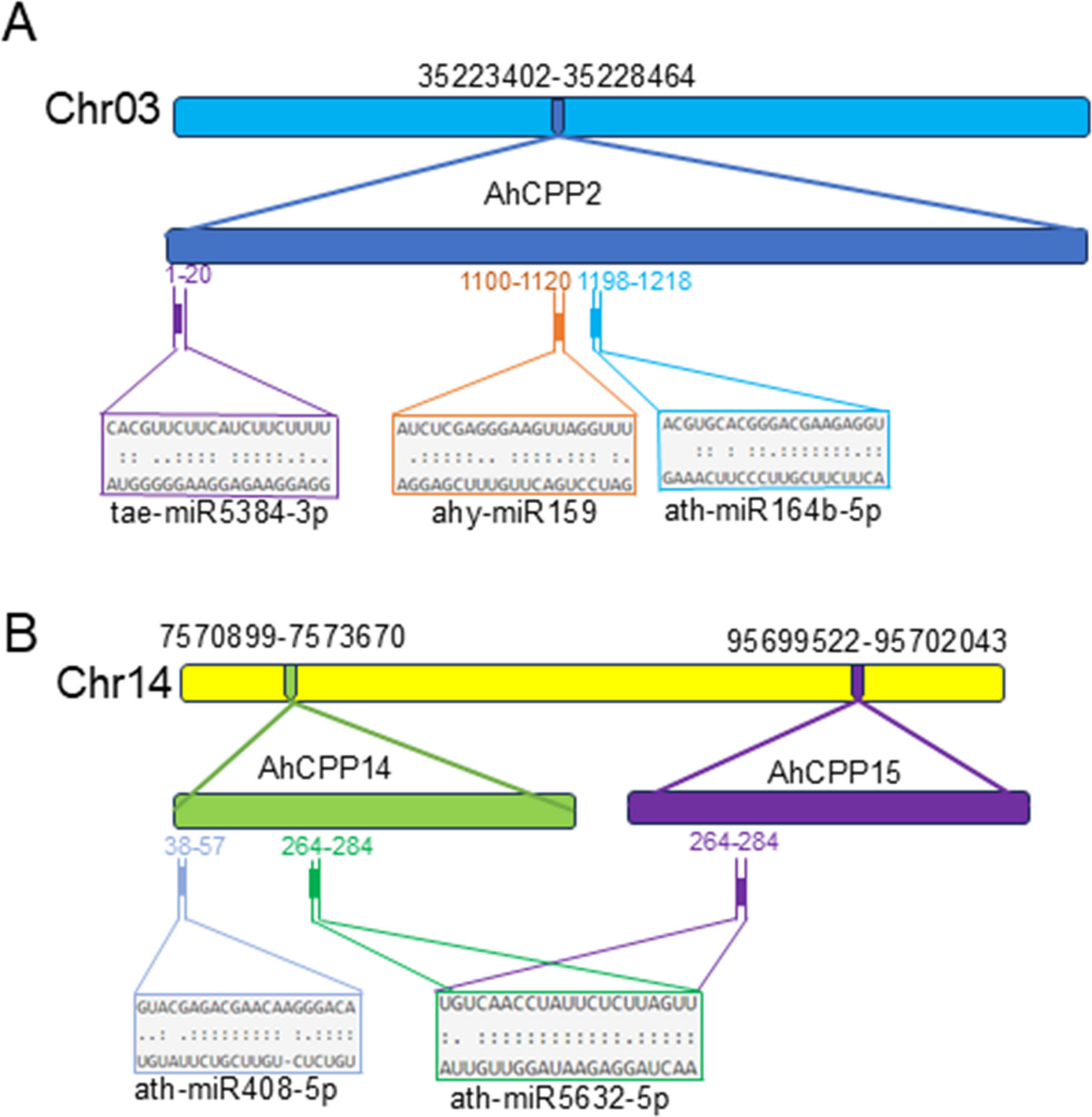
Schematic diagram of miRNA target sites in some AhCPP genes.

We identified 116 miRNAs (including 3 peanut-derived miRNAs) targeting 24 AhCPP genes through a comparative analysis between *Arabidopsis* and peanuts to investigate the miRNA-mediated posttranscriptional regulation of AhCPP genes (**Figure 6**; **Supplementary Table 6**). ath-miR5658 targeted the most genes (n=6), followed by ath-miR403-5p, ath-miR5012, ath-miR8181, ath-miR838, and ath-miR867 (4 targets each). The remaining miRNAs targeted 1–3 genes. Notably, several genes were regulated by multiple miRNAs: AhCPP2 (targeted by 14 miRNAs), AhCPP14 (2 miRNAs), and AhCPP15 (1 miRNA), with ath-miR5632-5p simultaneously targeting both AhCPP14 and AhCPP15. The miRNA binding sites for AhCPP2, AhCPP14, and AhCPP15 were mapped (**Figure 7**).

### Tissue-specific and stress-responsive expression patterns of AhCPP genes in peanut

**Figure 8:**
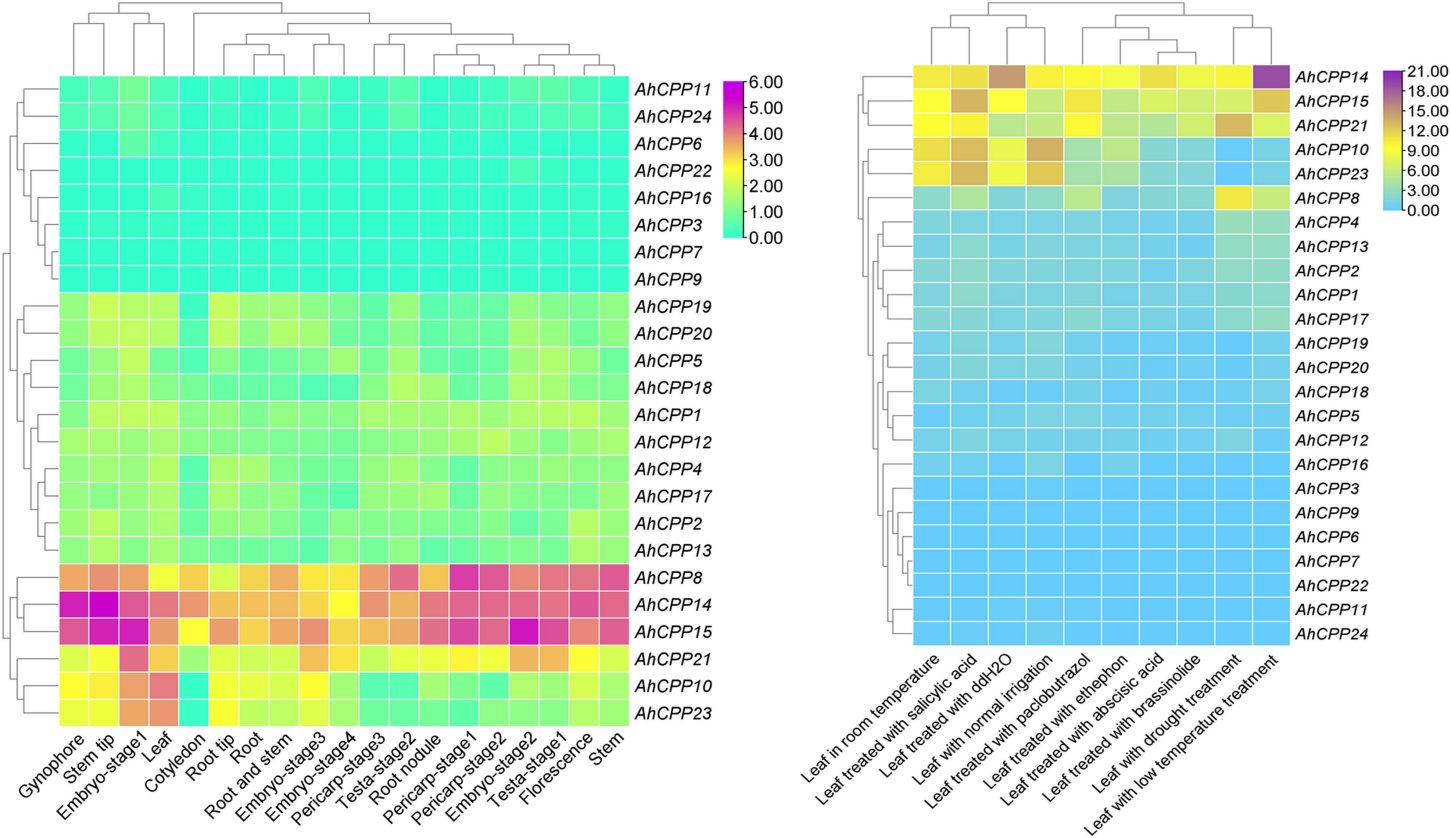
Heatmaps showing the expression of AhCPP genes under different conditions. **(A)** Expression in various peanut tissues. **(B)** Expression in response to different abiotic stresses. The hierarchical clustering analysis was performed using RNA-seq data.

An analysis of tissue-specific expression patterns facilitated the functional characterization of AhCPP genes in peanut. The distinct expression profiles of AhCPP family members indicate functional diversification. Notably, *AhCPP8*, *AhCPP14*, *AhCPP15*, and *AhCPP21* were relatively highly expressed in multiple tissues, particularly in stems, stem tips, embryos, root nodules, gynophores, testa, and florescence. *AhCPP10*, *AhCPP14*, *AhCPP15*, and *AhCPP23* were more highly expressed in leaves. High expression of *AhCPP8*, *AhCPP14*, *AhCPP15*, and *AhCPP21* in reproductive tissues (florescence and testa) indicates their essential functions in reproductive development and seed maturation. The functional diversity and tissue-specific expression patterns of AhCPP genes collectively ensure proper growth and physiological processes in peanut (**Figure 8A**; **Supplementary Table 7**). Transcriptome data were used to examine changes in the expression of the AhCPP genes in plants under drought, cold, ABA, brassinosteroid (BR), and ethephon stress and to investigate the involvement of these genes in abiotic stress responses (**Figure 8B**; **Supplementary Table 7**). Differential expression patterns indicated that the functions of the AhCPP members were unique during stress adaptation. *AhCPP14*, *AhCPP15*, and *AhCPP21* were markedly upregulated in stressed leaves, whereas *AhCPP14* was strongly upregulated in response to cold stress. *AhCPP8* was highly expressed in drought-stressed leaves. The consistently high expression of *AhCPP8*, *AhCPP14*, *AhCPP15*, and *AhCPP21* across both developmental and stress conditions implies that these genes play essential roles in abiotic stress tolerance and growth regulation.

### qRT–PCR verification of AhCPP transcriptional responses to abiotic stresses

Compared with normal plants, peanut seedlings subjected to drought and salt stress exhibited distinct morphological alterations that primarily manifested as significant changes in root length, root diameter, hypocotyl length, and plant height, with a particularly pronounced difference observed in hypocotyl elongation (**Supplementary Figure S5**).

**Figure 9:**
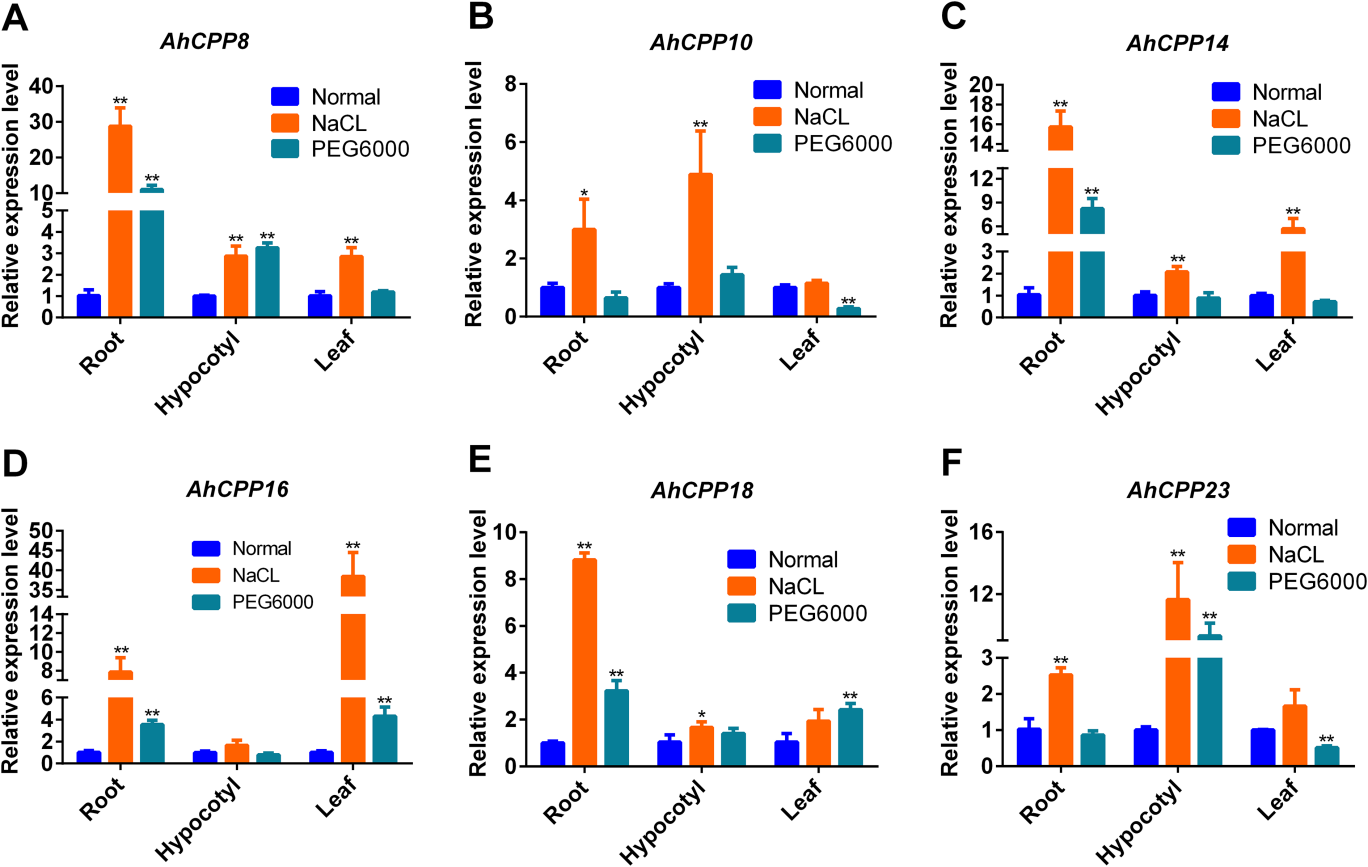
qRT–PCR analysis was performed to compare the relative expression levels of the six AhCPP genes in peanut roots, hypocotyls, and leaves under both normal and stress conditions. The x-axis depicts peanut tissues (root, hypocotyl, and leaf) under three conditions: normal conditions, salt stress, and drought stress. The y-axis shows the relative gene expression levels. The error bars represent the standard errors. Statistical significance is indicated as follows: *P < 0.05 and **P < 0.01.

Six genes were selected based on either their conserved collinearity with other species or their high expression levels according to RNA-seq data, and their dynamic changes in expression were analyzed during the peanut stress response to further validate the association between these phenotypic alterations and CPP genes at the genetic level. The results indicate that all AhCPP genes in peanuts respond to both salt stress and drought stress to varying degrees in the roots, hypocotyl, and leaves (**Figure 9**; **Supplementary Table 8**). Compared with that in normal roots, the expression of AhCPP8 was significantly increased in plants under stress, with a 28.07-fold increase observed following NaCl stress and a 10.73-fold increase observed following PEG stress. Similarly, this gene was highly significantly upregulated in hypocotyls under stress, whose expression levels were 2.87-fold (NaCl stress) and 3.26-fold (PEG stress) higher than those in normal hypocotyls. In leaves, its expression was significantly induced by salt stress (2.82-fold), whereas under drought stress, its expression tended to increase but the difference was not significant (**Figure 9A**). In plants under NaCl stress, the expression of *AhCPP10* was significantly upregulated in the roots and hypocotyls, reaching levels that were 2.98- and 4.86-fold higher than their respective normal levels, whereas no significant change was observed in the leaves. Additionally, in plants under PEG stress, this gene was significantly downregulated in leaves, whereas no significant alterations were detected in roots or hypocotyls (**Figure 9B**). NaCl stress induced the pronounced upregulation of the *AhCPP14* gene in all the examined tissues, with increases of 15.23-fold in the roots, 2.06-fold in the hypocotyls, and 5.66-fold in the leaves. In plants under drought stress, the expression of this gene was specifically upregulated in the roots (7.96-fold), whereas no significant changes were detected in the hypocotyls or leaves (**Figure 9C**). In contrast to the genes described above, the expression of *AhCPP16* remained unaltered in hypocotyls under both salt stress and drought stress. However, it was significantly altered in the roots and leaves, most strikingly showing a 38.14-fold upregulation in leaves under NaCl stress (**Figure 9D**). Compared with that in normal roots, *AhCPP18* expression was highly significantly upregulated in response to both salt (8.80-fold) and drought (3.22-fold) stress. Furthermore, an increasing trend was observed in hypocotyls and leaves in response to both stresses. Specifically, hypocotyls showed the significant upregulation of this gene under NaCl stress, whereas leaves exhibited highly significant upregulation of this gene under PEG stress (**Figure 9E**). *AhCPP23* displayed contrasting expression patterns in response to different abiotic stresses. During NaCl stress, it was upregulated in all the tissues, with the differences reaching significance in the roots (2.46-fold) and hypocotyls (11.60-fold). In contrast, in plants under PEG stress, its expression was extremely significantly upregulated in the hypocotyls (9.25-fold) but significantly downregulated in the leaves (0.51-fold), whereas its expression in roots showed a nonsignificant decreasing trend (**Figure 9F**).

## Discussion

Peanut is a vital allotetraploid crop that is cultivated for both oil and food (Bertioli et al., 2016). Although its tetraploid nature offers considerable genetic potential, the precise annotation and functional study of its genes are complicated by the large genome of this crop, which contains more than 80% repetitive sequences. Recent progress in sequencing technologies has led to the construction of a high-quality reference genome of approximately 2.54 Gb in size (Zhuang et al., 2019). This achievement, combined with the continuous enrichment of genomic resources, provides unprecedented opportunities for the systematic identification of gene families at the whole-genome level and for elucidating the genetic basis of key agronomic traits.

CPPs are known to be involved in plant growth, development, and stress responses (Hauser et al., 2000). Rapid advances in bioinformatics have enabled the genome-wide identification and characterization of CPP gene families across multiple species. Cultivated peanut is a globally important oilseed crop with typical tetraploid genome characteristics (Wang et al., 2025b), but systematic research on the CPP gene family in this crop is lacking because of the late completion of its genome sequence. The present study employed a genome-wide bioinformatics approach to identify 24 AhCPP genes within the cultivated peanut genome (**Table 1**). Physicochemical characterization revealed that with the exception of AhCPP14, all the AhCPPs were hydrophilic, which is consistent with findings reported in other species (Liu et al., 2025; Sun et al., 2023). AhCPP14 was identified as a hydrophobic protein containing two transmembrane domains and a CXC domain. These findings indicate that members of the CPP transcription factor family are not uniformly hydrophilic or hydrophobic and may perform distinct functions in different species (Sun et al., 2023).

Subcellular localization prediction revealed that all AhCPP proteins were localized to the nucleus (**Table 1**). This is consistent with observations in other species, including apple (Jiang et al., 2025), maize (Gu et al., 2025), *Lactuca sativa* (Zhu et al., 2025), Theaceae plants (Zheng et al., 2025), and wheat (Ullah et al., 2022), suggesting that nuclear localization may be a conserved feature of the CPP family. These proteins contain conserved cysteine-rich domains, such as the DNA-binding CXC domain. In *Arabidopsis*, Tesmin/TSO1-like CXC proteins act as transcriptional repressors, for example, by suppressing DNA methylation genes, often redundantly with paralogs such as TCX5 (Ning et al., 2020). These findings suggest that AhCPPs may similarly participate in transcriptional regulation, although this hypothesis requires experimental validation.

The AhCPP genes were unevenly distributed across all 20 chromosomes of peanut, with some chromosomes completely lacking these genes. This distribution pattern is consistent with that observed for CPP gene family members on chromosomes in other species, such as tomato and wheat (Sun et al., 2023; Ullah et al., 2022). Furthermore, the number of CPP genes identified varied among different species: 11 in rice and 8 in *Arabidopsis* (model plants), along with 8 in sorghum, 17 in moso bamboo, 12 in soybean, and 6 each in tomato and pigeon pea. In group 2, the clustering of the peanut genes AhCPP5, AhCPP8, and AhCPP18 with the tomato genes SlCPP1 and SlCPP2 was notable (**Figure 1**). These three AhCPP genes share highly similar motif compositions and gene structures, supporting their close relationships (**Figure 2**). The clustering of these genes within the same clade in the phylogenetic tree, despite the considerable phylogenetic distance between peanut (*Fabales*) and tomato (*Solanales*), suggests that AhCPP5, AhCPP8, AhCPP18, SlCPP1 and SlCPP2 are likely ancient orthologs. Notably, within the phylogenetic tree, the 19 AhCPPs in group 3 predominantly cluster with CPPs from soybean (*Glycine max*) and pigeon pea (*Cajanus cajan*), both leguminous plants (**Figure 1**). This clustering pattern may suggest that this group of genes has undergone a conserved evolutionary process following the divergence of the legume lineage and that their preservation may be important for biological processes specific to legumes. Moreover, AhCPP genes within the same subgroup exhibit structural similarity in their exon-intron organization, distribution of conserved motifs, and conserved domains, further supporting the inference of their functional similarity. For example, the arrangement of conserved protein motifs is identical or highly consistent between AhCPP2 and AhCPP13, as well as between AhCPP4 and AhCPP17 (**Figure 2**). Studies of *Arabidopsis* CPP genes have shown that *AtCPP4* (SOL1), which belongs to group 3- 2 in this study, is expressed in stamens and pollen mother cells and plays a critical role in floral organ development (Gu et al., 2025; Simmons et al., 2019). AhCPP10 and AhCPP23, which cluster in the same clade as AtCPP4 (**Figure 1**), also exhibit relatively high expression levels in florescence (**Figure 8**), suggesting that they may similarly be involved in regulating inflorescence differentiation in peanut.

Duplication events in the AhCPP gene family were analyzed based on the established role of genome duplication as a key driver of gene family expression, functional diversification, and neofunctionalization (Tsitsekian et al., 2019; Yang et al., 2022). The results revealed 27 duplicated gene pairs, with only one arising from tandem duplication and the remaining 26 attributable to segmental duplication. The Ka/Ks ratios of segmental duplication events were all less than 1, indicating that AhCPP genes underwent strong purifying selection during evolution (**Supplementary Figure S3**). Moreover, the collinearity analysis revealed substantial synteny and a considerable number of orthologous gene pairs between AhCPP genes and CPP genes in dicotyledonous plants (**Figure 4**), further supporting the evolutionary conservation and potential functional similarity of this gene family. These findings indicate that the CPP gene family exhibits a species-specific expansion pattern, as has also been reported for other gene families (Zhang et al., 2005).

Studies have shown that CPP gene promoters in various plants contain an abundance of cis-regulatory elements associated with developmental processes and abiotic stress tolerance (Bakhtari and Zamani, 2025; Tian et al., 2022; Zhu et al., 2025). Similarly, an analysis of the AhCPP gene promoters revealed an enrichment of cis-acting elements related to plant growth, development, and environmental stress tolerance. In addition to the cis-acting elements associated with biotic stress, abiotic stress, and light responses, numerous phytohormone-responsive elements, including ABREs, CGTCA motifs, TGACG motifs, TCA elements, TGA elements, and P-boxes, were also identified. Among the AhCPP genes, 19 of 24 members contain multiple ABREs in their promoter regions, with AhCPP18 harboring the greatest number (9 ABREs). As a pivotal signaling hormone in drought adaptation, abscisic acid (ABA) coregulates genes that harbor the conserved ABRE cis-element (PyACGTGG/TC) in their promoters, which is indispensable for activating transcription in response to ABA (Uno et al., 2000; Yamaguchi-Shinozaki and Shinozaki, 2005). These findings provide evidence that most AhCPP genes appear to be involved in drought stress responses through an ABA-dependent pathway, with AhCPP18 potentially serving as a key regulator of this process. Moreover, the presence of light−responsive regulatory elements (e.g., G−box and Sp1) in the promoter regions suggests that the AhCPP gene family may be broadly involved in plant light signal transduction. This observation is consistent with findings reported for the CPP gene families in *Gossypium hirsutum* (Huang et al., 2021) and Theaceae plants (Zheng et al., 2025). In peanut, the enrichment of these cis−acting elements within AhCPP gene promoters suggests their potential involvement in regulating both normal plant development and stress responses.

We investigated the miRNA-mediated posttranscriptional regulatory mechanisms targeting AhCPP genes in peanut by comparing *Arabidopsis* and peanut; we identified 116 miRNAs (including three peanut-derived miRNAs) targeting 24 AhCPP genes (**Figure 6**; **Supplementary Table 6**). miRNAs act as widespread posttranscriptional repressors in plants, directly suppressing the expression of target genes to regulate diverse biological processes (Rhoades et al., 2002). miRNAs and target genes form an intricate regulatory network capable of fine−tuning the expression of individual genes via multiple miRNAs or coordinating multiple genes through a single miRNA (Ding et al., 2020). Our predictions further support this hypothesis. Specifically, the results of the analyses revealed that *AhCPP2* is targeted by 14 miRNAs, *AhCPP14* by 2 miRNAs, and *AhCPP15* by a single miRNA, with ath-miR5632-5p simultaneously targeting both *AhCPP14* and *AhCPP15* (**Figure 7**). Additionally, ahy-miR3513-5p is predicted to target *AhCPP24* (**Figure 6**). Based on the established roles of miRNAs in plant development and stress adaptation, we hypothesized that these AhCPP-targeting miRNAs participate in crucial growth or environmental response pathways in peanut. This hypothesis requires experimental validation in future studies.

The transcriptomic analysis revealed that genes with constitutively high expression in multiple tissues were consistently upregulated under abiotic stress conditions (e.g., drought). This pattern was notably observed for *AhCPP8*, *AhCPP10*, *AhCPP14*, *AhCPP15*, *AhCPP21*, and *AhCPP23* (**Figures 8A, B**). The phenotypic analysis of peanut plants exposed to different stressors revealed that compared with the control treatment, both salt and drought stress significantly reduced the plant height (**Supplementary Figures S5A, B**). Consistent with these findings, studies of the morphological, physiological, and anatomical responses of peanut plants under drought stress have shown that under sustained drought conditions, morphological traits such as root length, shoot length, and plant height are reduced compared to control conditions, with the extent of the reduction depending on both the stress intensity and duration (Rani et al., 2019). Specifically, under our experimental stress conditions, the roots of plants subjected to drought stress were shorter and thicker than those of control plants. In contrast, salt-stressed roots tended to be slightly longer. Compared with that of unstressed plants, the hypocotyl length decreased significantly in response to both stress treatments. Furthermore, the leaf size was moderately smaller in the stressed plants. These phenotypic alterations may be linked to changes in the expression of CPP genes. According to the qRT–PCR results, the expression levels of *AhCPP8*, *AhCPP14*, *AhCPP16*, and *AhCPP18* in stressed roots were significantly higher than those in normal roots. In contrast, *AhCPP10* and *AhCPP23* expression was markedly increased in roots under salt stress, whereas their expression in roots under drought stress was reduced compared with than in normal roots, but the difference was not significant. These results suggest that members of the AhCPP gene family exhibit distinct transcriptional responses to salt and drought stress in root tissues. In *Phoebe bournei*, *PbCPP3* and *PbCPP4* were highly expressed in roots and stems, while other members showed limited expression, indicating tissue-specific regulatory roles (Liu et al., 2025). The consistently high basal expression of *AhCPP8*, *AhCPP14*, *AhCPP16*, and *AhCPP18* implies their potential involvement in root development or the maintenance of normal root physiology. The specific upregulation of *AhCPP10* and *AhCPP23* in plants under salt stress indicates that these two genes may be candidate regulators involved in the plant response to ionic stress. In comparison, the lack of significant changes in the expression these genes in response to drought stress suggests that *AhCPP10* and *AhCPP23* might respond more specifically to ionic stress than to osmotic stress. Similarly, TaCPP genes in wheat showed differential expression under heat, drought, and salt stress, and some members exhibited responses specific to roots and shoots (Ullah et al., 2022). These differential expression patterns may reflect differences in the transcriptional behavior of CPP transcription factors in response to different abiotic stresses, and the specific regulatory mechanisms involved warrant further investigation. Studies examining gene expression in two common bean cultivars have revealed that salt stress generally upregulates CPP gene expression, whereas drought stress selectively activates only a subset of CPP genes, with little to no effect on the expression of other members (Rakhimzhanova et al., 2023). This expression pattern is consistent with the behavior of the AhCPP genes observed in the present study. All qRT-PCR samples were collected concurrently with the phenotypic observation samples shown in **Supplementary Figure 5** at 5 days post-treatment, ensuring that the gene expression data reflect the transcriptional state when phenotypic differences emerged. This temporal consistency supports analyses of associations between gene expression and phenotype; however, establishing causality will require subsequent functional validation experiments.

An analysis of gene expression in the hypocotyl revealed that the expression of all the AhCPP genes tended to increase following salt stress, with the expression of all the genes except *AhCPP16* showing significant or highly significant induction. Under drought stress, the expression of *AhCPP8* and *AhCPP23* increased substantially compared with that in the control, whereas the expression of *AhCPP10* and *AhCPP18* showed a nonsignificant increasing trend. In contrast, the expression of AhCPP14 and *AhCPP16* tended to decrease, although the changes were not statistically significant (**Figure 9**). These patterns suggest that AhCPP family members exhibit distinct changes in expression in the hypocotyl following exposure to different stresses. The widespread upregulation in plants under salt stress implies the possible involvement of these genes in conserved pathways in response to ionic stress. In contrast, drought stress elicited a more complex expression profile: the pronounced upregulation of *AhCPP8* and *AhCPP23* indicated that these genes may be candidate drought−responsive genes, whereas the modest upregulation of *AhCPP10* and *AhCPP18* could be secondary or context−dependent expression patterns. The trends of decreased *AhCPP14* and *AhCPP16* expression may suggest their suppression or participation in alternative regulatory networks under drought conditions. This differential expression highlights the expression specificity and potential coordination among AhCPP genes in hypocotyl stress responses, offering clues for a further dissection of their regulatory mechanisms (**Figure 9**). During the seedling stage of wheat, TaCPP genes exhibit similar expression patterns in response to drought and salt stress (Ullah et al., 2022).

Interestingly, the expression patterns of these genes in leaves closely mirrored those observed in roots. For example, under salt stress, all the AhCPP genes were generally upregulated in the leaves, consistent with their expression pattern in the roots. This salt-responsive upregulation in leaf tissues has also been observed in other species. In tomato, salt stress induced distinct temporal responses among SlCPP genes, with *SlCPP6* peaking at 2 h at levels 4.15-fold higher than the control (Sun et al., 2023). Similarly, in *Phoebe bournei*, both salt and drought stress upregulated PbCPP genes in leaves (Liu et al., 2025). In contrast, under drought stress, the expression of these genes in leaves also differed: the expression of *AhCPP16* and *AhCPP18* was substantially upregulated, whereas that of *AhCPP10* and *AhCPP23* was significantly downregulated (**Figure 9**). These findings suggest that AhCPP genes may be subject to similar transcriptional regulatory mechanisms in different tissues in response to the same stress, particularly in the case of salt stress, which likely activates conserved signaling pathways operating throughout the plant (Ma et al., 2025). However, the differential expression patterns of certain genes in response to drought stress, such as *AhCPP10* and *AhCPP23*, which did not change significantly in the roots but was markedly decreased in the leaves (**Figures 9B, F**), may reflect tissue−specific regulatory networks or differences in the perception, transmission, or integration of stress signals across organs. In sorghum (Du et al., 2025), SbCPP genes exhibited dynamic expression patterns under saline-alkali stress. These findings provide a foundation for future functional characterization of AhCPP genes in peanut stress responses and developmental processes.

## Conclusions

In this study, we systematically identified 24 AhCPP genes in the cultivated peanut genome and performed a comprehensive analysis of their physicochemical properties, phylogenetic relationships, gene structures, duplication events, synteny, cis-regulatory elements, putative miRNA targets, and expression profiles across various tissues and following hormone and abiotic stress treatments. Additionally, the secondary and tertiary structural features of the encoded proteins were examined. Gene duplication events and Ka/Ks ratios indicated that this gene family has undergone strong purifying selection during evolution. The comparative synteny analysis across multiple species revealed widespread collinearity of AhCPP genes between monocots and dicots, with *AhCPP5* and *AhCPP18* maintaining syntenic relationships in all the species examined, highlighting their notable evolutionary conservation. An analysis of cis-regulatory elements and miRNA targets suggested that AhCPP genes are subject to multilayered regulation. Transcriptomic data illustrated the expression patterns of AhCPP genes in different tissues and under abiotic stresses, and the results of qRT−PCR further highlighted these genes as candidate regulators in the responses to drought and salt stress. Admittedly, determining the exact regulatory roles of individual AhCPP genes will require further functional validation in future studies. Collectively, these results establish a solid foundation for further elucidating the functional mechanisms of AhCPP genes in peanut stress resistance and provide valuable candidate gene resources for molecular breeding.

## Data Availability

The datasets presented in this study can be found in online repositories. The names of the repository/repositories and accession number(s) can be found in the article/**Supplementary Material**.
