# Supplementary material for: Comprehensive analysis of the cysteine-rich polycomb-like protein (CPP) gene family in peanut: insights into its expression patterns in abiotic stress responses
Source: Front Plant Sci. 2026 Apr 17;17:1799353. doi: 10.3389/fpls.2026.1799353 (PMC13132734; doi:10.3389/fpls.2026.1799353)
Supplement: Supplementary file 1 [file Presentation1.pptx]

## Slide 1
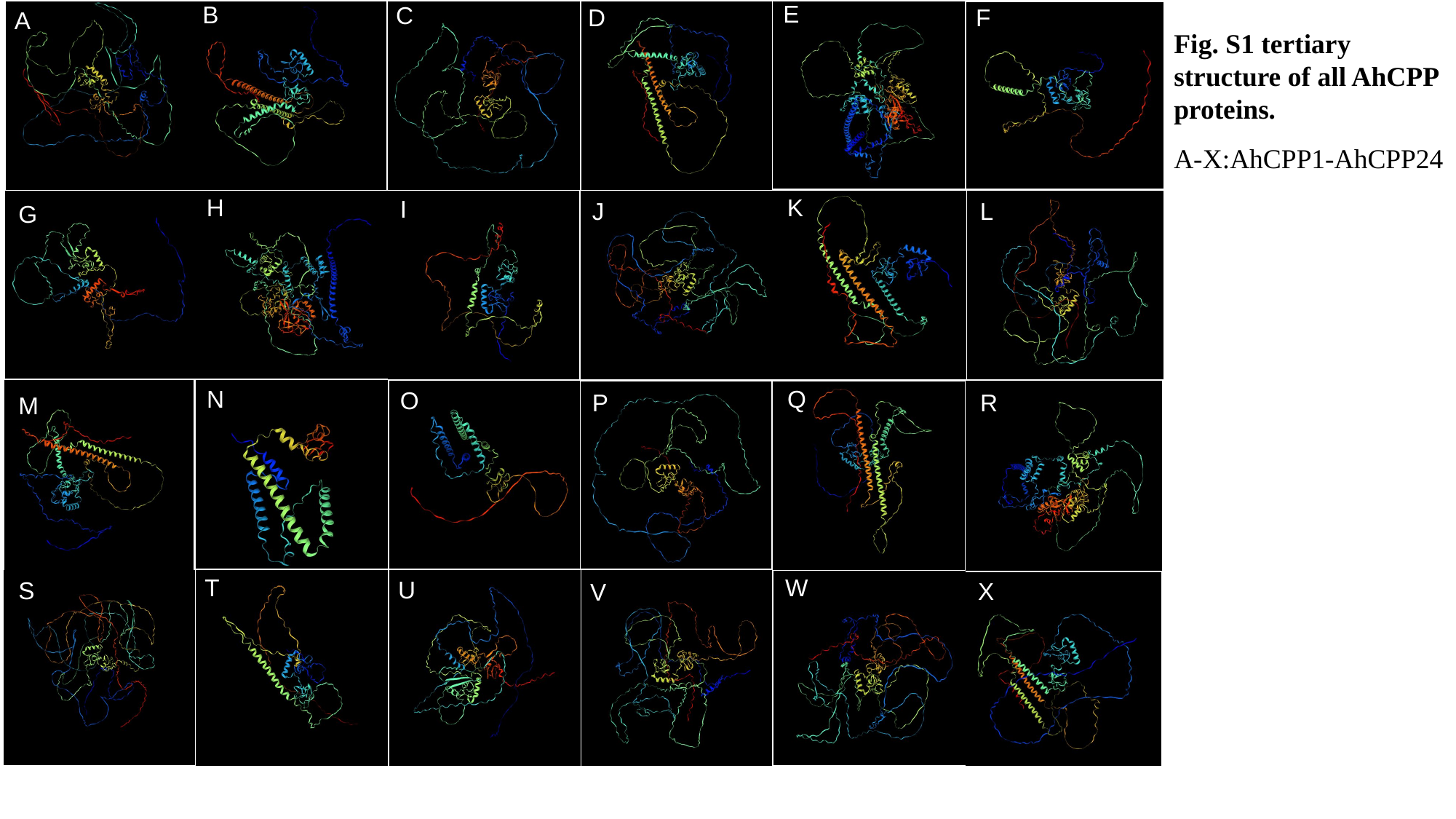

E
B
C
F
D
A
Fig. S1 tertiary structure of all AhCPP proteins.
A-X:AhCPP1-AhCPP24
K
H
I
L
J
G
Q
N
O
R
P
M
W
T
U
S
X
V

## Slide 2
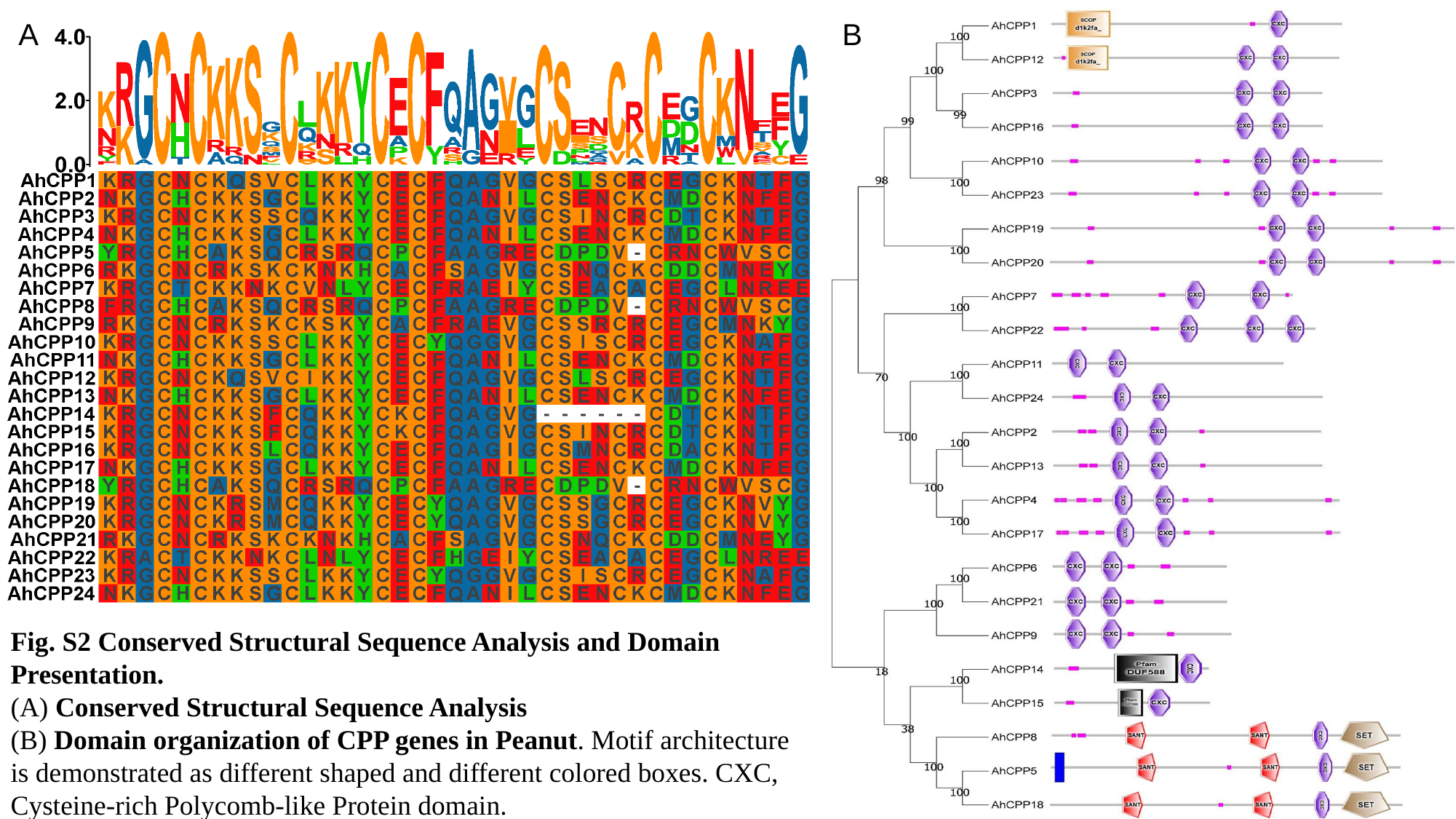

A
B
Fig. S2 Conserved Structural Sequence Analysis and Domain Presentation.
(A) Conserved Structural Sequence Analysis
(B) Domain organization of CPP genes in Peanut. Motif architecture is demonstrated as different shaped and different colored boxes. CXC, Cysteine-rich Polycomb-like Protein domain.

## Slide 3
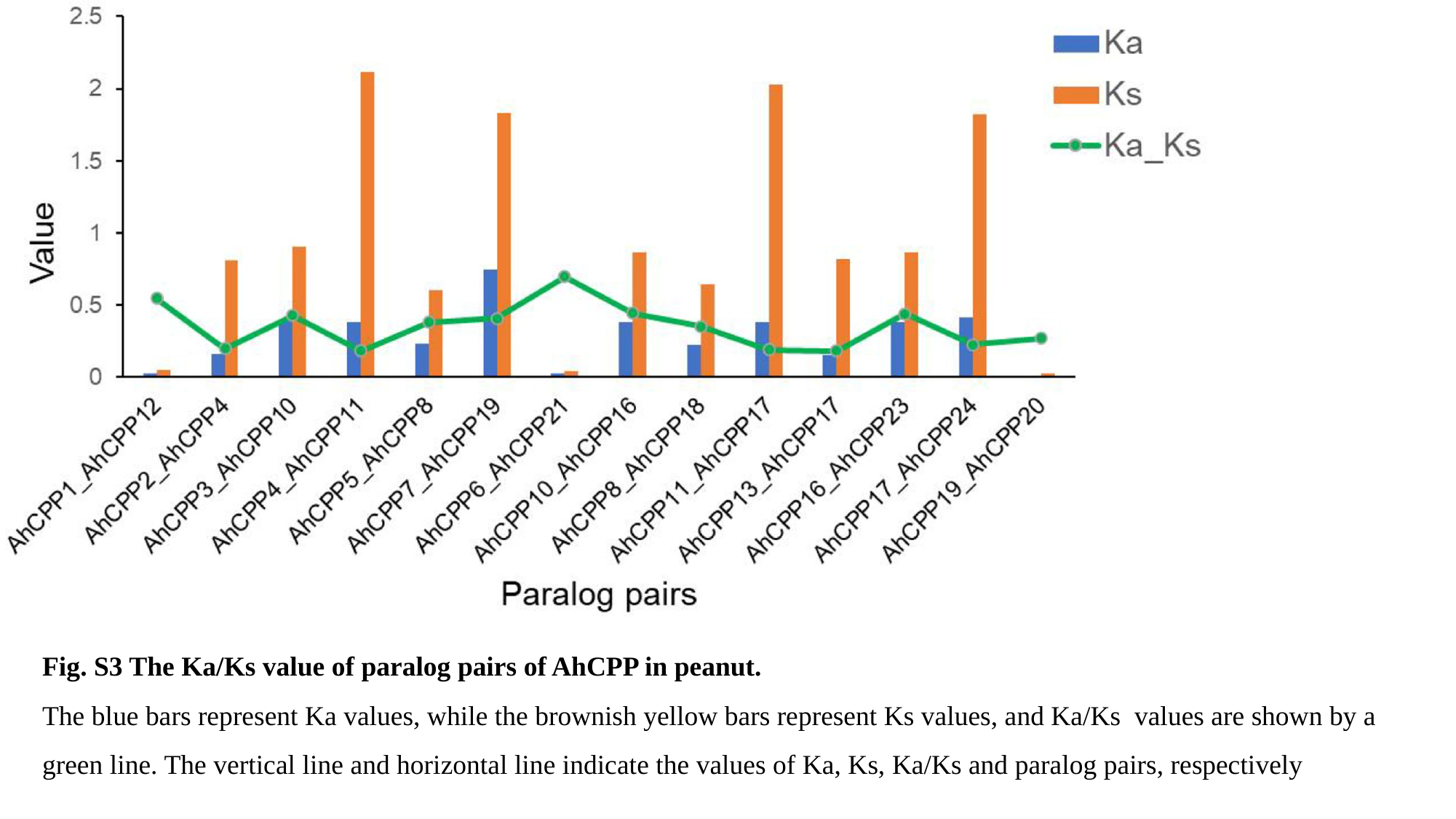

Fig. S3 The Ka/Ks value of paralog pairs of AhCPP in peanut.
The blue bars represent Ka values, while the brownish yellow bars represent Ks values, and Ka/Ks values are shown by a green line. The vertical line and horizontal line indicate the values of Ka, Ks, Ka/Ks and paralog pairs, respectively

## Slide 4
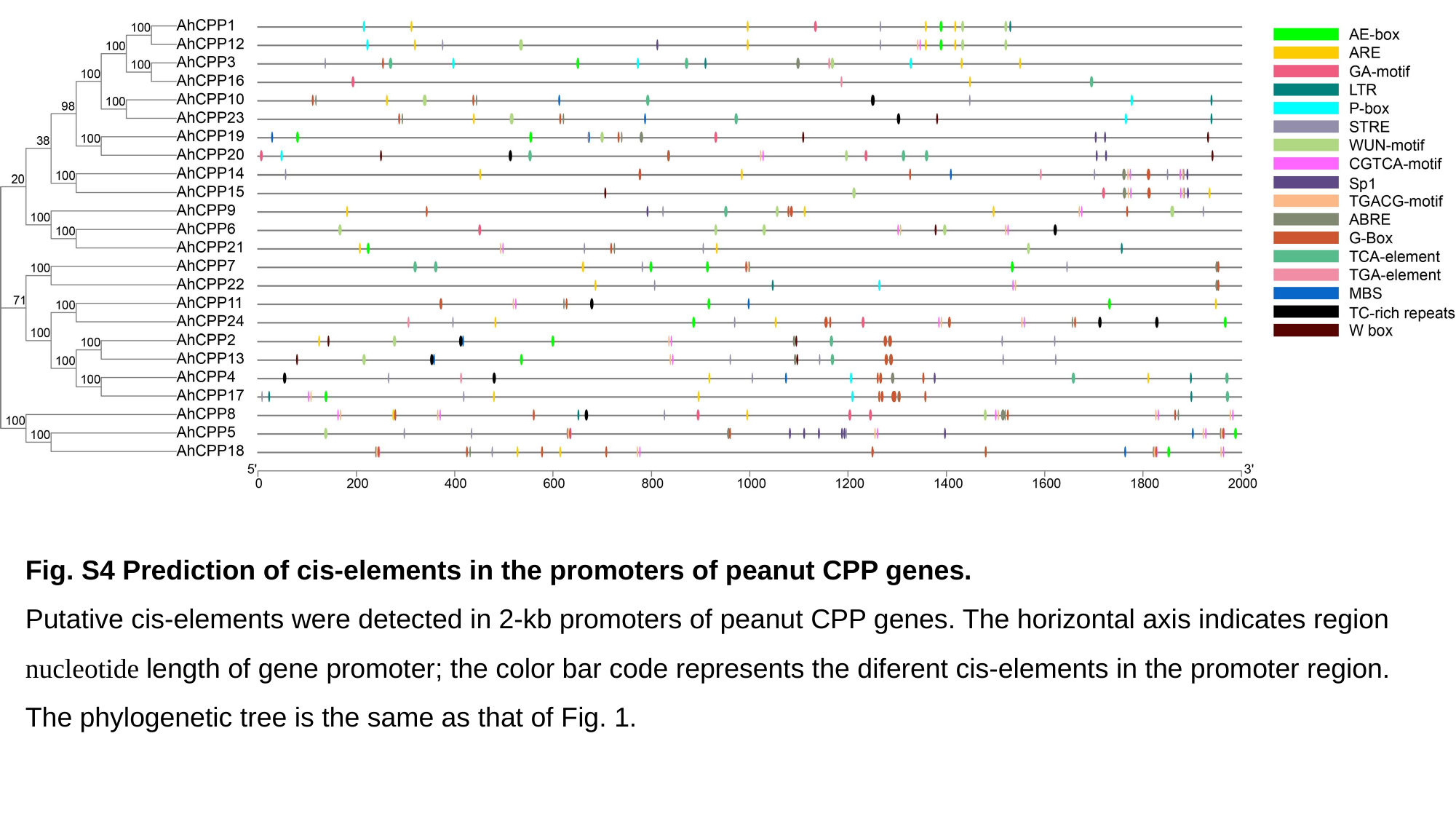

Fig. S4 Prediction of cis-elements in the promoters of peanut CPP genes.
Putative cis-elements were detected in 2-kb promoters of peanut CPP genes. The horizontal axis indicates region nucleotide length of gene promoter; the color bar code represents the diferent cis-elements in the promoter region. The phylogenetic tree is the same as that of Fig. 1.

## Slide 5
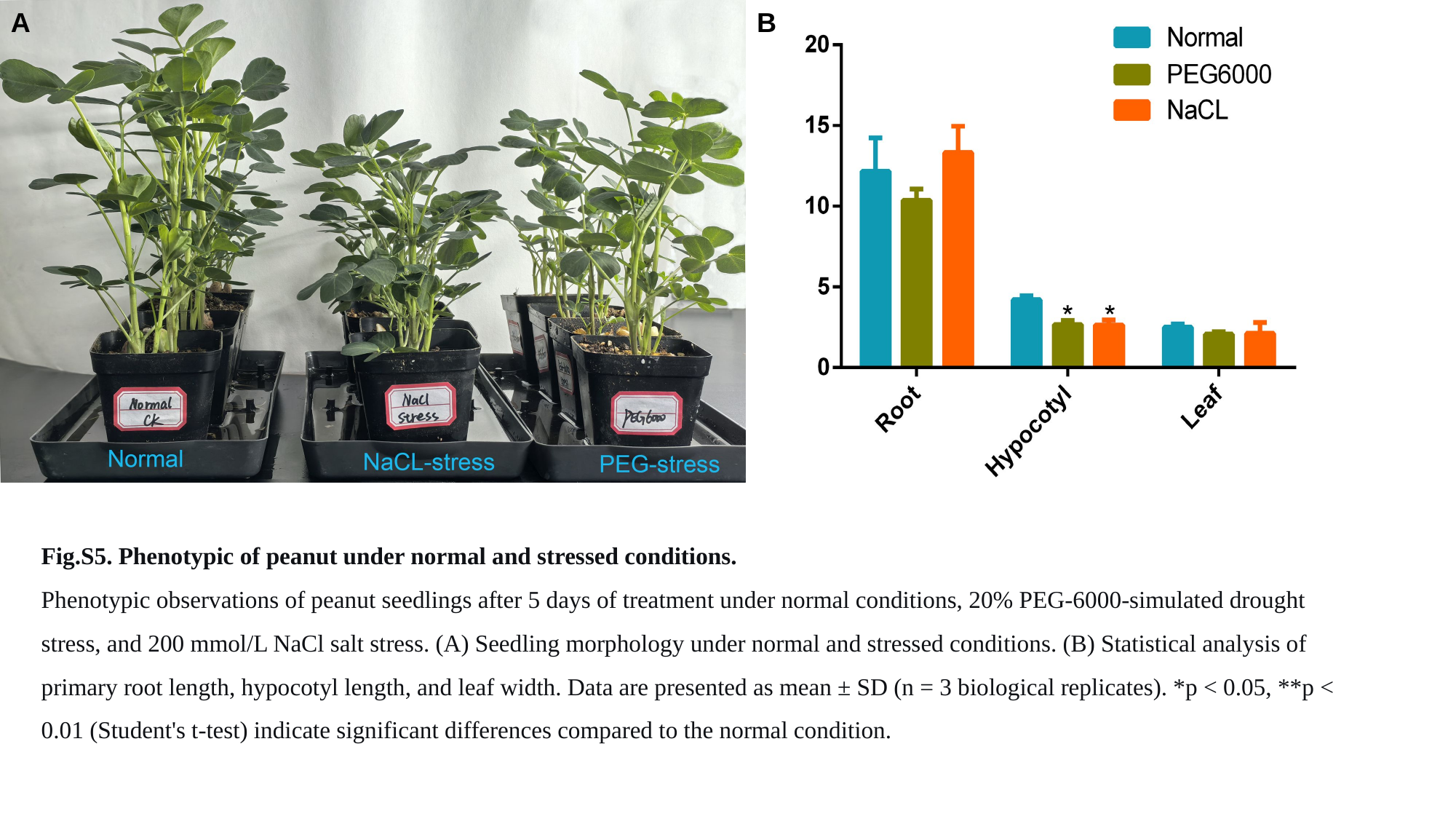

A
B
Fig.S5. Phenotypic of peanut under normal and stressed conditions.
Phenotypic observations of peanut seedlings after 5 days of treatment under normal conditions, 20% PEG-6000-simulated drought stress, and 200 mmol/L NaCl salt stress. (A) Seedling morphology under normal and stressed conditions. (B) Statistical analysis of primary root length, hypocotyl length, and leaf width. Data are presented as mean ± SD (n = 3 biological replicates). *p < 0.05, **p < 0.01 (Student's t-test) indicate significant differences compared to the normal condition.
